# Ionic and cellular mechanisms underlying TBX5/PITX2 insufficiency-induced atrial fibrillation: Insights from mathematical models of human atrial cells

**DOI:** 10.1038/s41598-018-33958-y

**Published:** 2018-10-23

**Authors:** Jieyun Bai, Patrick A. Gladding, Martin K. Stiles, Vadim V. Fedorov, Jichao Zhao

**Affiliations:** 1 Auckland Bioengineering Institute, The University of Auckland, Auckland, New Zealand; 2 School of Computer Science and Technology, Harbin Institute Technology, Harbin, China; 3 Department of Cardiology, Waitemata District Health Board, Auckland, New Zealand; 4 Waikato Hospital, Hamilton, New Zealand; 5 Department of Physiology and Cell Biology, The Ohio State University Wexner Medical Center, Columbus, United States of America

## Abstract

Transcription factors TBX5 and PITX2 involve in the regulation of gene expression of ion channels and are closely associated with atrial fibrillation (AF), the most common cardiac arrhythmia in developed countries. The exact cellular and molecular mechanisms underlying the increased susceptibility to AF in patients with TBX5/PITX2 insufficiency remain unclear. In this study, we have developed and validated a novel human left atrial cellular model (TPA) based on the ten Tusscher-Panfilov ventricular cell model to systematically investigate how electrical remodeling induced by TBX5/PITX2 insufficiency leads to AF. Using our TPA model, we have demonstrated that spontaneous diastolic depolarization observed in atrial myocytes with TBX5-deletion can be explained by altered intracellular calcium handling and suppression of inward-rectifier potassium current (*I*_*K1*_). Additionally, our computer simulation results shed new light on the novel cellular mechanism underlying AF by indicating that the imbalance between suppressed outward current *I*_*K1*_ and increased inward sodium-calcium exchanger current (*I*_*NCX*_) resulted from SR calcium leak leads to spontaneous depolarizations. Furthermore, our simulation results suggest that these arrhythmogenic triggers can be potentially suppressed by inhibiting sarcoplasmic reticulum (SR) calcium leak and reversing remodeled *I*_*K1*_. More importantly, this study has clinically significant implications on the drugs used for maintaining SR calcium homeostasis, whereby drugs such as dantrolene may confer significant improvement for the treatment of AF patients with TBX5/PITX2 insufficiency.

## Introduction

Atrial fibrillation (AF), the most common cardiac arrhythmia, causes substantial mortality, morbidity and impaired quality of life in an aging population^1^. Current clinical treatment of AF is suboptimal, as a single one-size-fits-all treatment formula is used regardless of the different preconditions that lead to AF^2^. AF is a complex disease and known to be associated with multiple risk factors including hypertension, obesity^2^, diabetes mellitus^3^, ischemic heart disease, heart failure and stroke^2,3^. Recent human genome-wide association studies (GWAS) suggest that AF is also a heritable disease^4^. Over the past decade, some common genetic variants underlying AF in the general population, e.g., a T-box transcription factor TBX5 and a paired-like homeodomain transcription factor 2 PITX2, have been discovered^5–14^.

TBX5 plays important roles in heart development and cardiac rhythm control^15^. GWAS have discovered that the lack of TBX5 is associated with abnormalities in action potential, which leads to an increased risk of developing AF^16^. Various research laboratories have demonstrated that TBX5 potentially regulates AF through the modulation of multiple ion-channel genes, suggesting that electrical remodeling is a major contributor to cellular arrhythmogenic triggers^17–20^. In addition, the loss of function of PITX2 was observed in the TBX5-deletion atria, signifying that PITX2 takes part in TBX5 insufficiency induced-AF^21^. Multiple studies have revealed that PITX2 on human 4q25 locus is associated with AF where reduced PITX2 promotes an arrhythmogenic substrate^22–26^. Pro-arrhythmogenic effects of PITX2 are twofold: 1) it regulates the expression of genes involved in ion channels^27^, leading to increased electrical-remodeling-related risk of AF^28^, and 2) since PITX2 plays a crucial role in left-right atrial asymmetry during cardiac development^29^, PITX2-deletion can cause malformation of the pulmonary veins^30^, which play a substantial role in initiating and maintaining spontaneous AF^31^. Therefore, the reduced function of PITX2 in TBX5-deleted atrial myocytes may also contribute to AF, and collectively, electrical remodeling induced by the insufficiency of PITX2 and TBX5 may be a major contributor of the increased susceptibility to AF^32–35^.

How electrical remodeling, regulated by the transcription regulation network governed by TBX5 and modulated by PITX2, contributes to AF has not been thoroughly investigated. Computational models^36^ provide a powerful tool for assessing the impact of individual remodeled ion currents on the action potential (AP) and calcium dynamics which is not possible in experimental and clinical studies. To achieve this, in this study a robust computer model of human atrial cellular kinetics (TPA) based on the ten Tusscher-Panfilov (TP) ventricular cell model^37^ was developed, validated, and utilized to systematically illustrate the cellular and molecular mechanisms underlying spontaneous AF in patients with TBX5/PITX2 insufficiency.

## Results

### Simulation of TBX5 insufficiency-induced phenotype

**Figure 1:**
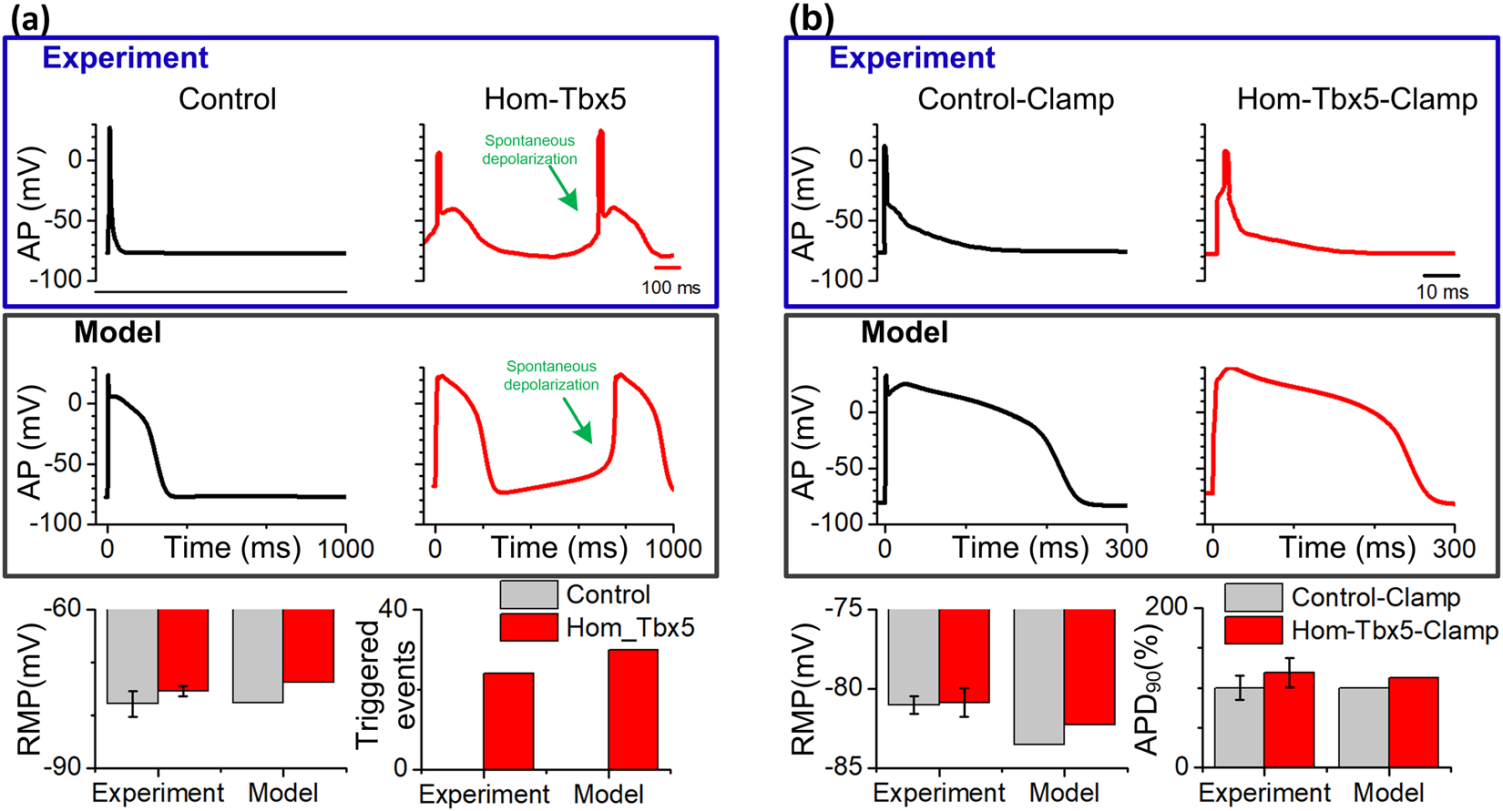
Simulated action potential (AP) in human atrial cells and experimental AP in mouse atrial cells. (**a**) Comparison between APs from our human atrial model (Model) and those from the experimental adult mouse heart by Nadadur *et al*.^21^ under control (black) and homozygous TBX5-knockout (Hom-Tbx5; red) conditions. Representative abnormal depolarization events (i.e., spontaneous depolarization is indicated by the arrow) were observed in mouse atrial myocytes and reproduced using our human atrial cell model. Compared to control atrial cells, the resting membrane potential (RMP) and the number of triggered events were increased under the Hom-Tbx5 condition. (**b**) The simulated APs using our human atrial cell model were compared to representative APs of mouse atrial myocytes under control (Control-Clamp) and Hom-Tbx5 (Hom-Tbx5-Clamp) conditions. Triggered activity in Hom-Tbx5 atrial myocytes was suppressed under the Hom-Tbx5-Clamp condition. RMP and action potential duration (APD_90_) were increased, compared to Control-Clamp. This figure demonstrates that our human atrial model is capable of reproducing experimentally obtained AP characteristics of the Hom-Tbx5 atrial myocytes.

To determine whether spontaneous depolarizations can be induced in the homozygous TBX5-knockout (Hom-Tbx5) human atrial myocytes, computer simulations were conducted using our human atrial TPA model under both control and Hom-Tbx5 models at a pacing frequency of 1 Hz. Experimental AP recordings of murine atrial myocytes^21^ and simulated AP profiles using the TPA model are shown in Fig. 1(a). Mild AP prolongation and spontaneous depolarizations are all evident in Hom-Tbx5 atrial myocytes (red) but absent from control myocytes (black). Regarding the characteristics of AP, resting membrane potential (RMP) increased from −77.62 mV (Control) to −73.85 mV (Hom-Tbx5) and the number of triggered events jumped from 0 to 30/30 (Hom-Tbx5). These results are consistent with the experimental findings reported by Nadadur *et al*.^21^ by carefully studying atrial myocytes isolated from Hom-Tbx5 versus control mice, where their reported RMP and the number of triggered beats were −75.47 ± 0.94 mV versus −77.88 ± 2.4 mV and 24/30 versus 0, respectively (bottom panel of Fig. 1(a)). Our computer simulation results support the concept that electrical remodeling induced by TBX5 insufficiency can lead to triggered activity in human atrial cells.

To confirm the hypothesis that the cytosolic calcium concentration ([*Ca*^2+^]_i_) is involved in TBX5 insufficiency-induced triggered activity, [*Ca*^2+^]_i_ in the Hom-Tbx5 model was set to a steady value (0.07 µM) based on data collected in an experimental study^21^. Figure 1(b) shows the suppression of spontaneous depolarizations in human Hom-Tbx5 atrial cells by clamping [*Ca*^2+^]_i_ observed in an experimental^21^ (black) and our modeling (red) study. Our computer simulation results are consistent with experimental observations that RMP increased from −83.51 mV (Control-Clamp) to −82.30 mV (Hom-Tbx5-Clamp) and action potential duration at 90% repolarization (APD_90_) was prolonged by 12.7% (from 232.9 ms to 262.5 ms). The comparison showed that the Hom-Tbx5 model has obtained AP characteristics similar to those from TBX5-mutant atrial myocytes observed in experimental studies^21^.

### Ionic basis of TBX5 insufficiency induced phenotype

**Figure 2:**
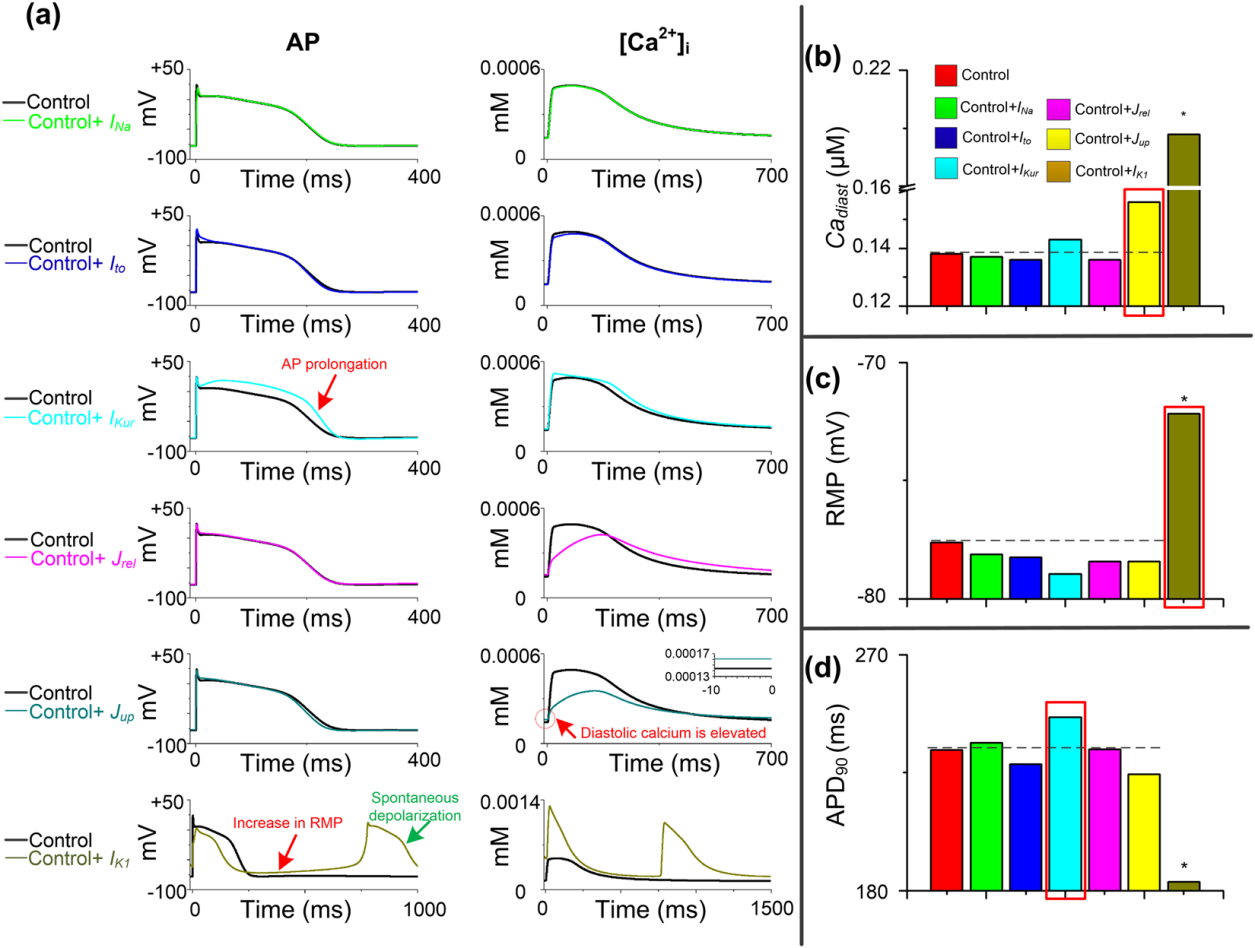
Effects of individual ionic remodeling targets on the cytosolic calcium concentration ([*Ca*^2+^]_i_) and action potential (AP). (**a**) Characteristics of control atrial cells with each TBX5-remodeled cellular component i.e., fast sodium current, *I*_*Na*_; transient outward potassium current, *I*_*to*_; ultrarapid delayed rectifier potassium current, *I*_*Kur*_; calcium flow through the sarcoplasmic reticulum calcium ATPase (SERCA), *J*_*up*_, calcium flow through the ryanodine receptor (RyR), *J*_*rel*_ and inward rectifier potassium current, *I*_*K*1_, respectively. Spontaneous depolarizations are induced in human atrial cells with the remodeled *I*_*K*1_. AP prolongation, diastolic calcium elevation and increase in resting membrane potential (RMP) occur in human atrial cells with remodeled *I*_*Kur*_, *J*_*up*_ and *I*_*K*1_, respectively. (**b**–**d**) Diastolic calcium concentration (*Ca*_*diast*_), RMP and action potential duration (APD_90_) for all cell variants are compared to control atrial myocytes (red bar). Main components which contribute to AP abnormalities in Hom-Tbx5 atrial cells are marked with red rectangles. Biomarkers of spontaneous depolarizations are marked with stars.

Hom-Tbx5-induced electrical remodeling, including fast sodium current *I*_*Na*_, transient outward potassium current *I*_*to*_, ultrarapid delayed rectifier potassium current *I*_*Kur*_, calcium flow through the sarcoplasmic reticulum calcium ATPase (SERCA) *J*_*up*_, calcium flow through the ryanodine receptor (RyR) *J*_*rel*_, and inward rectifier potassium current *I*_*K*1_, caused AP prolongation, abnormal autonomous depolarization and elevated diastolic [*Ca*^2+^]_i_. To evaluate the contribution of each individual remodeled ion current to these APs and *Ca*^2+^ handling abnormalities, we have conducted a series of computer simulations by incorporating each individual ionic remodeling into the control model (Fig. 2(a)). We then systematically compared the diastolic calcium concentration (*Ca*_*diast*_), RMP and APD_90_ under different conditions (Fig. 2(b–d)). Compared with atrial control cells, spontaneous depolarizations were induced in the control model with *I*_*K1*_ remodeling, but not under other remodeling conditions. Prolonged APD, increased RMP and elevated *Ca*_*diast*_ were observed in the control model with remodeled *I*_*Kur*_, *I*_*K1*_, and *J*_*up*_, respectively.

**Figure 3:**
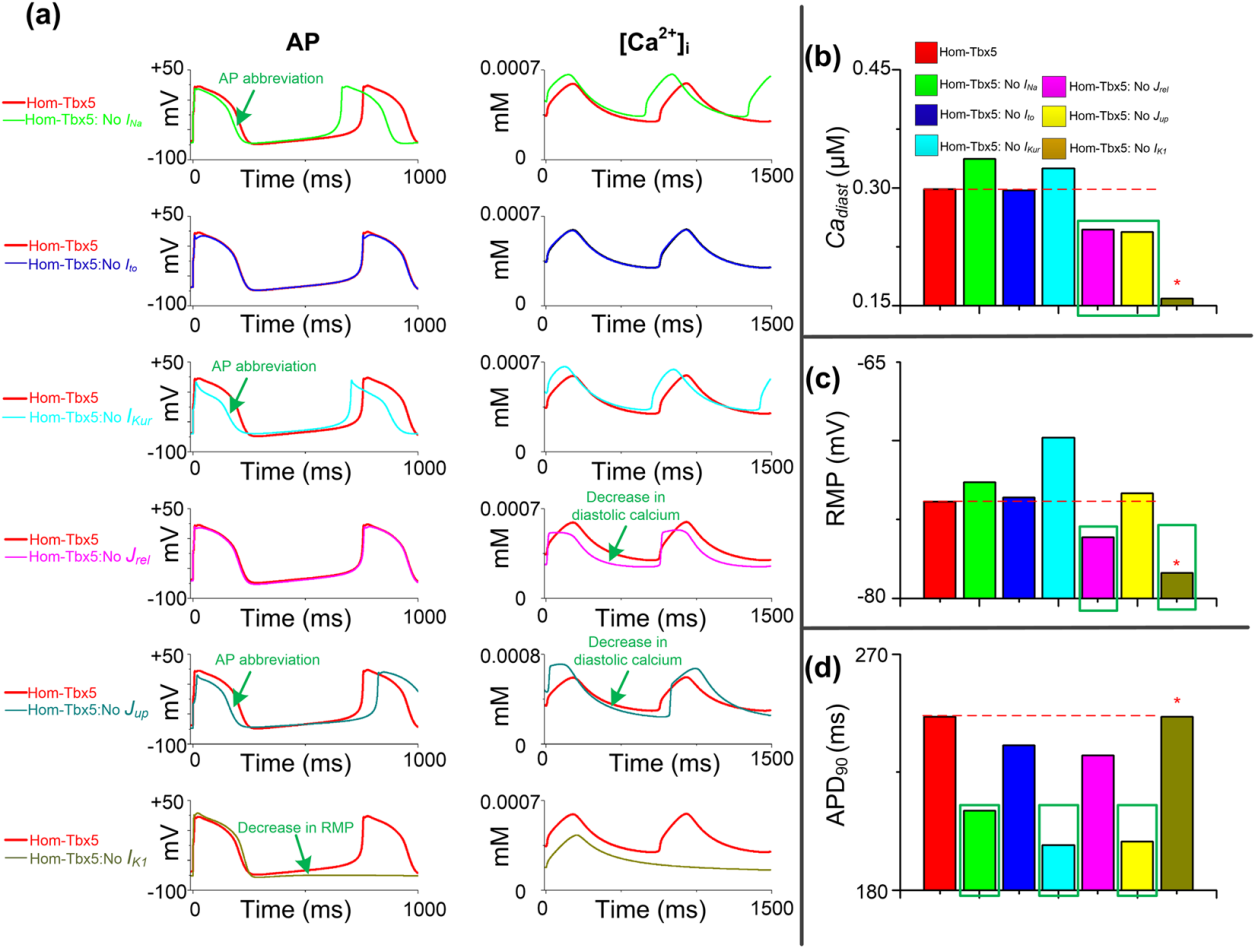
Effects of reversing remodeling of individual ionic channels on the cytosolic calcium concentration ([*Ca*^2+^]_i_) and action potential (AP). (**a**) Characteristics of homozygous TBX5-knockout (Hom-Tbx5) atrial cells without each remodeled cellular component, i.e., *I*_*Na*_, *I*_*to*_, *I*_*Kur*_, *J*_*up*_, *J*_*rel*_ and *I*_*K*1_, respectively. Spontaneous depolarizations were suppressed in human atrial cells without the remodeled *I*_*K*1_. Action potential duration (APD_90_) was well restored when the remodeling of *I*_*Na*_, *I*_*Kur*_ and *J*_*up*_ were individually reversed. Diastolic calcium was decreased in Hom-Tbx5 atrial cells without effects of the remodeled *J*_*rel*_ and *J*_*up*_. (**b**–**d**) The diastolic calcium concentration (*Ca*_*diast*_), resting membrane potential (RMP) and APD_90_ for all cell variants were compared to those of Hom-Tbx5 atrial myocytes (red bar). Main components which contribute to AP abnormalities in Hom-Tbx5 atrial cells are marked with green rectangles. Biomarkers of the normal action potential are marked with stars.

To further explore the putative targets among the remodeled cellular components that contribute to the Hom-Tbx5 phenotype, we have conducted a series of computer simulations with modified Hom-Tbx5 models by reversing each remodeled ionic component separately and analyzed the characteristics of the resultant APs and calcium transient (Fig. 3). Compared with spontaneous depolarizations of Hom-Tbx5 atrial cells, reversing *I*_*K1*_ remodeling rescued spontaneous depolarizations in Hom-Tbx5 atrial cells, but not under other conditions (Fig. 3(a)), which re-emphasizes the important role of *I*_*K1*_ in the atrial pathology. Furthermore, the three biomarkers, i.e., *Ca*_*diast*_, RMP and APD_90_, were systematically analyzed. Compared with Hom-Tbx5 atrial cells, *Ca*_*diast*_ was well restored by reversing *J*_*rel*_ and *J*_*up*_ independently (Fig. 3(b)), indicating that both *J*_*rel*_ and *J*_*up*_ contribute to abnormalities in the [*Ca*^2+^]_i_ of Hom-Tbx5 atrial cells. In addition to the remodeled *I*_*K1*_, RMP was also reduced by excluding effects of remodeled *J*_*rel*_ (Fig. 3(c)), suggesting that disrupted calcium flow involves in abnormal cellular depolarizations of Hom-Tbx5 atrial cells. Reversing remodeled *I*_*Na*_, *I*_*to*_, *I*_*Kur*_, *J*_*up*_ and *J*_*rel*_ separately caused a substantial reduction in APD_90_, but not under the remodeled *I*_*K1*_ (Fig. 3(d)). These results suggest that disrupted calcium fluxes, i.e., *J*_*rel*_ and *J*_*up*_, are responsible for AP abnormalities which include AP prolongation and abnormal autonomous depolarization in Hom-Tbx5 atrial myocytes.

### Role of PITX2 in TBX5 insufficiency-induced phenotype

**Figure 4:**
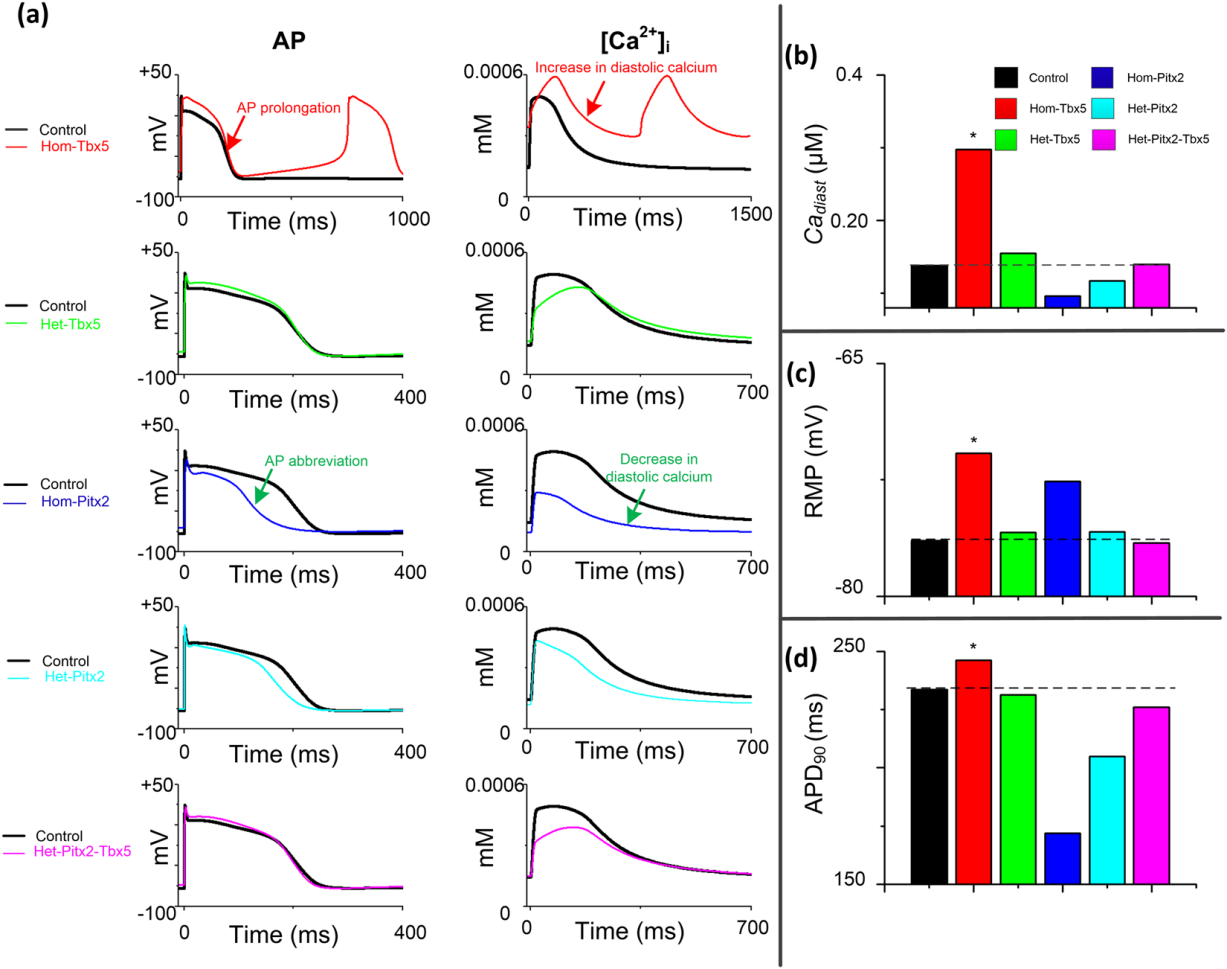
Role of the TBX5-PITX2 regulatory loop in spontaneous depolarization generation. (**a**) Cytosolic calcium concentration ([*Ca*^2+^]_i_) and action potential (AP) characteristics in homozygous TBX5-knockout (Hom-Tbx5), heterozygous TBX5-knockout (Het-Tbx5), homozygous PITX2-knockout (Hom-Pitx2), heterozygous PITX2-knockout (Het-Pitx2) and heterozygous knockout of both PITX2 and TBX5 (Het-Pitx2-Tbx5) atrial cells. TBX5 insufficiency (e.g., Hom-Tbx5) led to AP prolongation and diastolic calcium elevation, whereas PITX2 insufficiency (e.g., Hom-Pitx2) caused AP abbreviation and a decrease in diastolic calcium. AP abnormalities induced by TBX5 haploinsufficiency were rescued by PITX2 haploinsufficiency. (**b**–**d**) The diastolic calcium concentration (*Ca*_*diast*_), resting membrane potential (RMP) and action potential duration (APD_90_) for all cell variants were compared to control atrial myocytes (black bar).

Previous studies have shown that the most important human AF locus PITX2 is significantly down-regulated in the TBX5-mutant atria^21^. To investigate the role of PITX2 in the development of triggered activity in Hom-Tbx5 atrial cells, computer simulations were conducted with Hom-Tbx5, heterozygous TBX5-knockout (Het-Tbx5), homozygous PITX2-knockout (Hom-Pitx2), heterozygous PITX2-knockout (Het-Pitx2) and heterozygous knockout of both PITX2 and TBX5 (Het-Pitx2-Tbx5) conditions (for details see Methods) and the effects of TBX5/PITX2 interplay on APs and [*Ca*^2+^]_i_ were examined (Fig. 4). For TBX5-knockout atrial cells, the Hom-Tbx5 model had more abundant *Ca*_*diast*_ and longer APD_90_, compared with the control model. However, APD_90_ reduction and low *Ca*_*diast*_ were observed in PITX2-knockout atrial cells (including Hom-Pitx2 and Het-Pitx2), in comparison to the control condition. For heterozygous knockout of both PITX2 and TBX5 atrial cells, i.e., Het-Pitx2-Tbx5, APD_90_ and *Ca*_*diast*_ were comparable to those of the control condition (Fig. 4(a)). Figure 4(b–d) show a detailed comparison of all the biomarkers, i.e., *Ca*_*diast*_, RMP and APD_90_, between the control APs and those of remodeling APs, demonstrating antagonistic effects on APs between TBX5 insufficiency and PITX2 insufficiency. These results support experimental observations that TBX5 and PITX2 antagonistically regulate membrane effector genes and reduced PITX2 due to TBX5 insufficiency rescues atrial gene expression abnormalities^21^.

### Potential antiarrhythmic effects of dantrolene in TBX5-insufficiency cardiomyocytes

**Figure 5:**
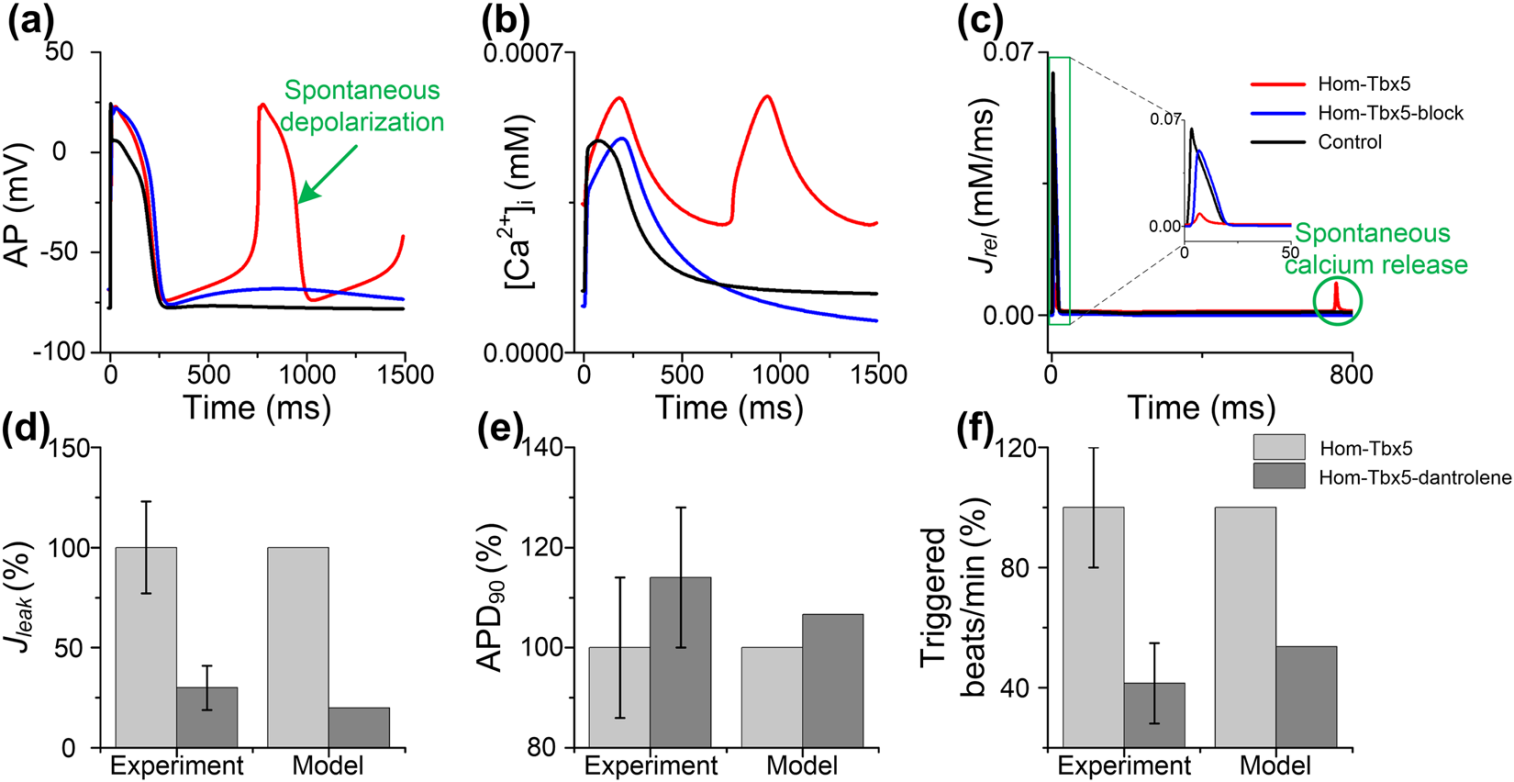
Role of sarcoplasmic reticulum (SR) calcium leak (*J*_*leak*_) in spontaneous depolarization generation. Simulated action potential (AP) (**a**), cytosolic calcium concentration [*Ca*^2+^]_i_ (**b**) and calcium flow *J*_*rel*_ (**c**) through the ryanodine receptor (RyR) are displayed under control, homozygous TBX5-knockout (Hom-Tbx5) and Hom-Tbx5 with inhibition of *J*_*leak*_ (Hom-Tbx5-block). According to the effect of dantrolene on *J*_*leak*_ (**d**), when *J*_*leak*_ was inhibited by 80% (Hom-Tbx5-dantrolene), changes in APD_90_ (**e**) and triggered beats/min (**f**) compared to Hom-Tbx5 are shown. Simulated results were compared to the experimental data by Hartmann *et al*.^38^.

Dantrolene is a drug used to stabilize RyR for the treatment of malignant hyperthermia. Previous studies have shown that dantrolene beneficially influences disrupted sarcoplasmic reticulum (SR) calcium homeostasis by inhibiting SR calcium leak (*J*_*leak*_) in atrial myocytes from patients with AF^38^. To evaluate the role of *J*_*leak*_ in TBX5 insufficiency-induced arrhythmias, we have conducted computer simulations in which *J*_*leak*_ was blocked in Hom-Tbx5 atrial cells. As shown in Fig. 5(a–c), when *J*_*leak*_ was completely inhibited, spontaneous depolarizations, disrupted [*Ca*^2+^]_i_ and diastolic *J*_*rel*_ frequency in Hom-Tbx5 atrial cells were decreased. However, APD_90_ and RMP of the Hom-Tbx5-block model were not well restored compared with the control model. These data suggest that SR calcium leak partly contributes to TBX5 insufficiency-induced arrhythmias.

Dantrolene (10 µM) significantly reduced total SR calcium leak of human AF cardiomyocytes by 56% ± 28%. To examine whether dantrolene has antiarrhythmic effects on spontaneous depolarizations induced by TBX5 insufficiency, we conducted simulations in which *J*_*leak*_ was blocked by 80% (Hom-Tbx5-dantrolene) to mimic the treatment with dantrolene (10 µM) in left human atrial cardiomyocytes from patients with AF (Fig. 5(d)). Simulated results were then compared to the experimental data reported by Hartmann *et al*.^38^. As shown in Fig. 5(e,f), under the Hom-Tbx5-dantrolene condition, APD_90_ was elongated by 6.6% and the frequency of the triggered events was reduced by 46.4%, compared to the Hom-Tbx5 condition. Importantly, simulated changes in *J*_*leak*_, APD_90_ and triggered events/min were within the range of experimental data observed by Hartmann *et al*.^38^, suggesting that dantrolene may be a potential antiarrhythmic drug for patients with TBX5 insufficiency.

## Discussion

Many studies over the past several decades have demonstrated that transcription factors, e.g., TBX5 and PITX2, can directly or indirectly influence atrial rhythm by regulating the expression of membrane effector genes^5,13^. However, the exact mechanisms, particularly how electrical remodeling induced by these transcription factors contribute to the increased susceptibility to AF, remain unclear due to its nature of complexity and limitation of clinical/experimental studies^39^. To our knowledge, this is the first study in silico to show that TBX5 insufficiency-induced electrical remodeling predicts the incidence of triggered activity in human atrial cells and lends support to the concept of a crucial pathophysiological role of TBX5 insufficiency in the development of AF.

Based on the TP model of ventricular epicardial cells^37^, we developed a human atrial cellular model, i.e., TPA, to use in our study by taking into account ionic differences between atrial and ventricular cells^40,41^. The calcium transient and AP profile of the TPA model are comparable to the Grandi *et al*. model^40^ and Courtemanche *et al*. model (CRN)^42^, respectively. The APD restitution curve of the TPA model is more accurate compared with experimentally measured data by Bosch *et al*.^43^. Most importantly, modifications to calcium-induced calcium release for reproducing early afterdepolarization (EAD)^44^ and delayed afterdepolarization (DAD) (Supplementary Fig. S4)^45^ were included in the TPA model. Our model also produces spontaneous depolarizations observed in mouse atrial myocytes obtained from the TBX5-deletion atria^21^. In addition, the modified CRN model (CRN_TP), which was developed by combining calcium handling of the TPA model with the transmembrane currents of the CRN model (Supplementary Fig. S7), can reproduce DAD due to SR calcium leak and spontaneous depolarization due to a reduction of *I*_*K1*_ (Supplementary Fig. S8), indicating the powerful tool for simulating human atrial electrophysiology. Therefore, the TPA model for quantitatively describing human atrial cellular kinetics may be a powerful tool for investigating arrhythmic behaviors (i.e., EAD, DAD and spontaneous depolarizations) in human atrial cells.

**Figure 6:**
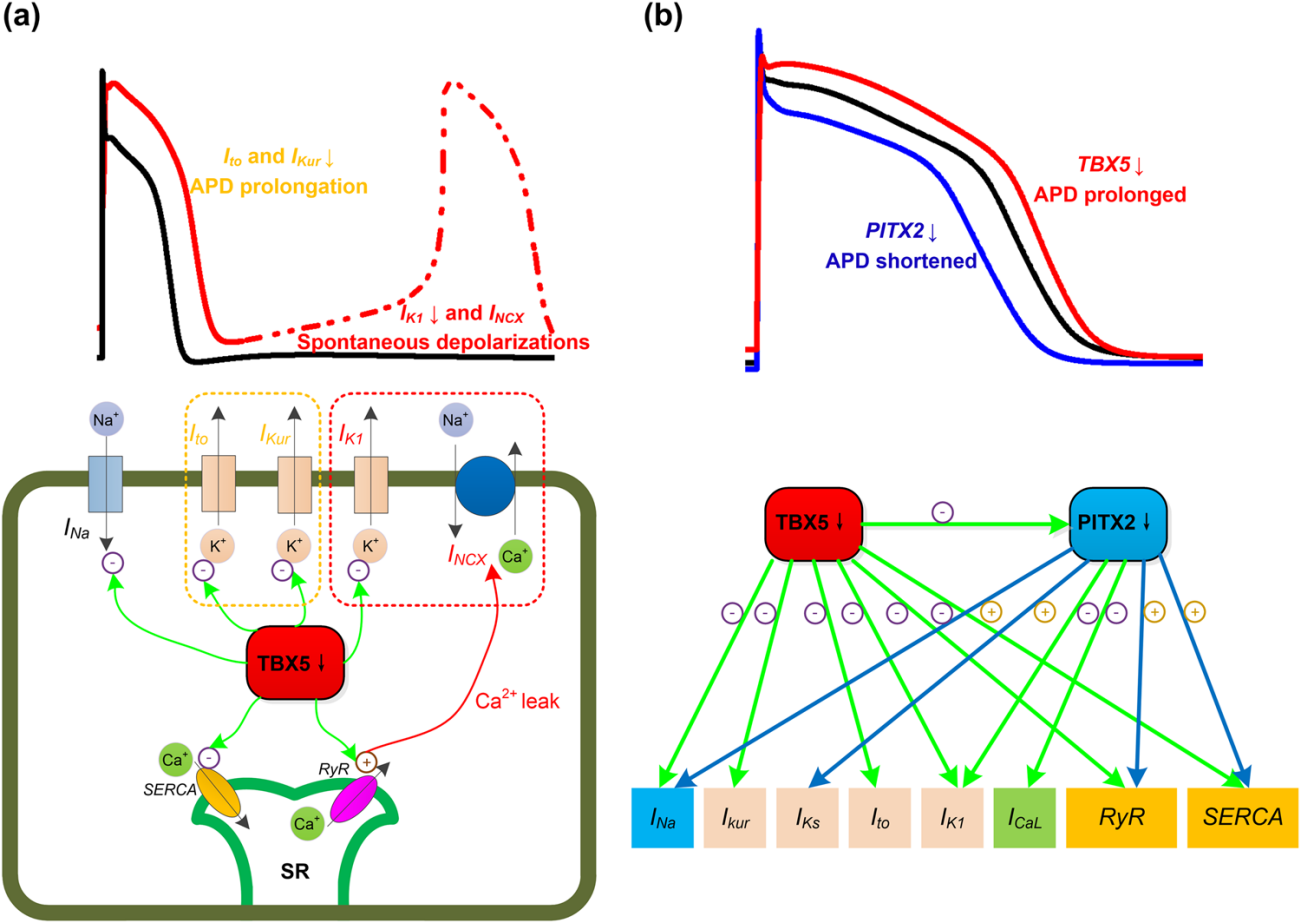
The role of electrical remodeling of TBX5 insufficiency in atrial fibrillation. (**a**) The schematic illustration of the impact of TBX5 loss-of-function mutation on ionic currents/action potential duration (APD). TBX5-deletion leads to reductions in *I*_*Na*,_ *I*_*to*_, *I*_*Kur*_, *I*_*K1*_, SERCA and RyR. Reduced repolarizing potassium currents (e.g., *I*_*to*_ and *I*_*Kur*_) lead to prolonged APD. Suppression of *I*_*K1*_ and increased *I*_*NCX*_ due to cytosolic calcium overload lead to phase 4 depolarization and predispose to spontaneous depolarizations. (**b**) TBX5 regulates PITX2 expression, and TBX5 and PITX2 antagonistically regulate downstream targets. Reduced PITX2 leads to upregulation of *I*_*Na*,_ *I*_*Ks*_, SERCA and RyR, and downregulation of *I*_*CaL*_ and *I*_*K1*_. Loss of TBX5 leads to prolonged APD, whereas loss of PITX2 leads to shortened APD. Therefore, reduced PITX2 in TBX5-mutant atria contributes to a protective mechanism.

Computer simulation results in this study have demonstrated that diastolic SR calcium release promotes the development of spontaneous depolarizations, contributing to triggered activity in AF patients with TBX5 insufficiency. Experimental studies have indicated that calcium handling remodeling produces a vulnerable substrate for AF maintenance, suggesting that increased diastolic SR calcium leak and related delayed afterdepolarizations/triggered activity promote cellular arrhythmogenesis in AF patients^46–48^. Some studies of the effect of TBX5 mutants on calcium handling also suggest that abnormal diastolic SR calcium release is a crucial arrhythmogenic trigger^21,49^. Adult specific TBX5-deletion leads to significant down-regulation of numerous genes (e.g., SERCA) required for calcium handling^21^, suggesting that SR calcium release is impaired in atrial cells of homozygous TBX5-knockout atria (Fig. 6(a)). Treating TBX5-deletion atrial myocytes with the calcium-chelator 1, 2-bis (o- aminophenoxy) ethane-N, N, N′, N′-tetraacetic acid (BAPTA) reversed AP abnormalities (i.e., AP prolongation and abnormal depolarizations), which indicates that disrupted calcium handling is the main cause of TBX5 insufficiency-induced AF^21^. In an experimental study of the adult-specific TBX5-knockout mice^49^, the authors observed reductions in SR calcium load and the rate of SR calcium uptake, and an increase in *I*_*NCX*_ in atria, and hence they concluded that calcium extrusion via *I*_*NCX*_ provides a molecular mechanism for afterdepolarizations (Fig. 6(a)). In our study, we have further discovered that diastolic SR calcium level of TBX5- knockout atrial myocytes is increased and blocking SR calcium leak rescues TBX5 insufficiency-induced spontaneous depolarizations. In addition to using our TPA cell model, we employed the Voigt *et al*. human atrial cell model^46^ to test the single cell predictions arising from TBX5 insufficiency and the simulated results showed that TBX5-induced electrical remodeling leads to decreased systolic [*Ca*^2+^]_i_, increased *Ca*_*diast*_ and increased calcium leak (Supplementary Figs S5 and S6). Furthermore, our TPA and CRN_TP models also predicted the development of DADs due to increased SR calcium leak (Supplementary Figs S4 and S8). These studies and our results suggest that increased SR calcium leak leads to the enhanced diastolic elimination of calcium ions via *I*_*NCX*_, which consequently causes TBX5 insufficiency-induced spontaneous depolarizations, and hence AF.

Our simulation results also demonstrate that the TBX5 insufficiency-induced reduction in *I*_*K1*_ leads to phase-4 depolarization and spontaneous depolarizations. Previous studies have assessed that the impact of the suppression of *I*_*K1*_ on non-autonomous cardiomyocytes, indicating that reduced *I*_*K1*_ contributes to spontaneous depolarizations^50–53^. Some experiments demonstrated that spontaneous depolarizations can be produced by genetic suppression of *I*_*K1*_ in ventricular myocytes^50^. Our computer modeling studies showed that the imbalance between suppressed outward *I*_*K1*_ and increased inward *I*_*NCX*_ causes slow phase-4 depolarization (Supplementary Figs S4 and S8), suggesting that spontaneous depolarizations are carried by *I*_*NCX*_ which depends on [Ca^2+^]_i_^51,52^. These aforementioned studies and our computer simulations provide evidence that suppression of *I*_*K1*_ contributes to elevated RMP and generation of spontaneous depolarizations by disrupting the imbalance between outward and inward currents. In TBX5-knockout atria, KCNJ2 (encoding for *I*_*K1*_) is significantly down-regulated and its precise role in AF is unclear^21^. In our study, the role of suppressed *I*_*K1*_ in the development of TBX5 insufficiency-induced spontaneous depolarizations was assessed. Our simulation results showed that spontaneous depolarizations can be induced by TBX5 insufficiency-remodeled *I*_*K1*_ and that reversing remodeled *I*_*K1*_ can rescue TBX5 insufficiency-induced spontaneous depolarizations (Fig. 6(a)). Thus, our data and other studies support the concept that *I*_*K1*_ is crucial for maintaining RMP and excitability, and suggest that suppression of *I*_*K1*_ contributes to TBX5 insufficiency-induced spontaneous depolarizations.

Recent studies have reported that PITX2 expression is reduced in TBX5-mutant mice^21^, while TBX5 gene expression is not significantly changed in PITX2 deficiency mice^54^. Experimental studies showed that PITX2 insufficiency-induced electrical remodeling includes upregulation of SCN5A (encoding for *I*_*Na*_)^21^, KCNQ1 (encoding for *I*_*Ks*_)^54,55^, RyR (encoding for *J*_*rel*_)^21,54,56^ and SERCA (encoding for *J*_*up*_)^21,54,56^, and downregulation of KCNJ2 (encoding for *I*_*K1*_)^57^ and CACNA1C (encoding for *I*_*CaL*_)^28,56^. On the other hand, TBX5 insufficiency-induced electrical remodelling includes reductions of *I*_*Na*_, *I*_*to*_, *I*_*Kur*_, *I*_*K1*_, RyR and SERCA^21^. Further studies demonstrated that TBX5 and PITX2 antagonistically regulate *I*_*Na*_, *J*_*rel*_ and *J*_*up*_^21^. Our results demonstrate that electrical remodeling induced by PITX2 insufficiency causes AP abbreviation, in line with the experimental observation in heterozygous PITX2 mice, linking PITX2 insufficiency to AF^28^. Simulated results from this study also showed that electrical remodeling caused by TBX5 insufficiency leads to APD prolongation and triggered activity, indicating AF susceptibility in the presence of TBX5 insufficiency. PITX2 insufficiency or TBX5 insufficiency causes opposite effects on the human atrial APD, yet both cause the increased susceptibility to AF (Fig. 6(b)). Additionally, our computer simulations also suggest that changes in APD caused by TBX5 insufficiency can be rescued by PITX2 insufficiency, explaining the role of the TBX5-PITX2 gene regulatory network for atrial rhythm control. These findings are consistent with the experimental observation of Nadadur *et al*.^21^ in atrial cells, in which the physiologic effects of reduced TBX5 dose, including decreased expression of critical AF genes, atrial rhythm instability, cellular AF abnormalities and AF susceptibility, were all rescued by reduced PITX2 dose. Our data and these studies suggest that the TBX5-driven PITX2-modulated incoherent feed-forward loop is important for maintaining atrial rhythm.

In patients with AF, impaired calcium handling (elevated diastolic [Ca^2+^]_i_) and afterdepolarizations are a common observation in cellular pathophysiology^46–48^. Here, we show that normal calcium dynamics is necessary for normal APs and suppression of diastolic SR calcium leak can rescue TBX5 insufficiency-induced spontaneous depolarizations. This may suggest that calcium extrusion caused by SR calcium leak via *I*_*NCX*_ favors phase-4 depolarization and triggered activity. Experimental data showed that dantrolene, a drug known to stabilize RyR, can decrease the frequency of calcium waves, diastolic SR calcium leak and spontaneous calcium release, and significantly suppress DADs in human AF cardiomyocytes^38^. Our simulation results suggest that dantrolene may beneficially influence disrupted calcium handling, specifically diastolic SR calcium leak, and prevent the occurrence of TBX5 insufficiency-induced spontaneous depolarizations.

**Figure 7:**
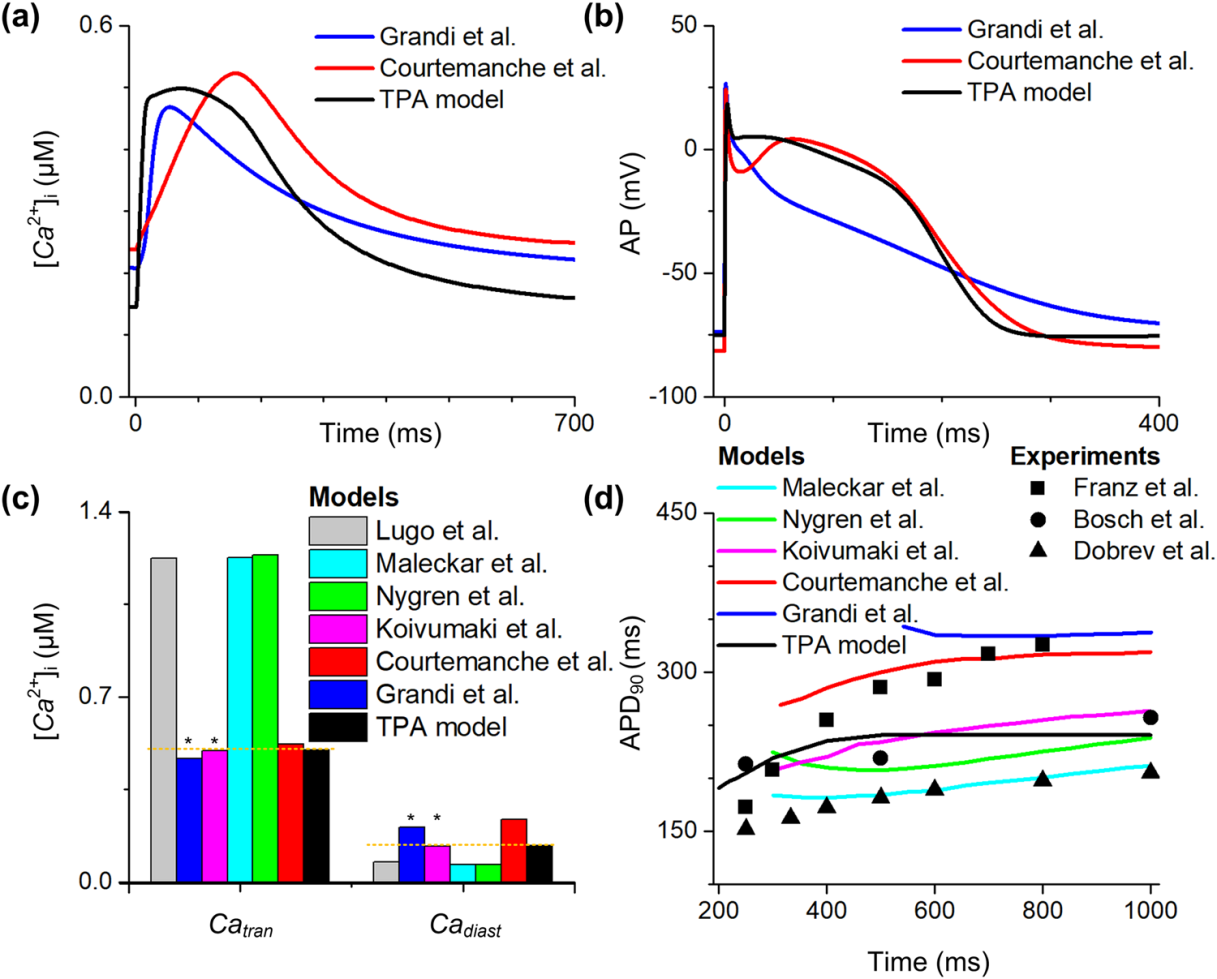
Comparison of the TPA model with different human atrial models. The myocyte was stimulated at a pacing frequency of 1 Hz. The intracellular calcium ([*Ca*^2+^]_i_, (**a**)) and action potentials (AP, (**b**)) of Grandi *et al*. model^40^ (blue lines), Courtemanche *et al*.model^42^ (red lines) and the current model (black lines) are shown. (**c**) The amplitude of calcium transient (*Ca*_*tran*_) and diastolic calcium concentration (*Ca*_*diast*_) (black bar) are compared to simulation results of Lugo *et al*. model (gray bar)^66^, Maleckar *et al*. model^67^ (cyan bar), Nygren *et al*. model^68^ (green bar), Koivumaki *et al*. model^64^ (magenta bar), Courtemanche *et al*. model (red bar) and Grandi *et al*. model (blue bar). (**d**) The action potential duration at 90% repolarization (APD_90_) restitution curve (black lines) is compared to simulated APD_90_ restitution of Maleckar *et al*. model (cyan lines), Nygren *et al*. model (green lines), Koivumaki *et al*. model (magenta lines), Courtemanche *et al*. model (red lines) and Grandi *et al*. model (blue lines), and experimental data of Franz *et al*.^69^ (▪), Bosch *et al*.^43^ (•) and Dobrev *et al*. (▲)^70^. All the data are adapted from Wilhelms *et al*.^71^.

Several limitations specific to this study are addressed here. Firstly, in the absence of the required human experimental data, when simulating effects of TBX5 insufficiency on ion channels, electrical remodeling was assumed to be identical in both human and animal atria, and characteristics of the simulated human APs were compared with experimental data of mouse cells for validation. These assumptions warrant further investigations and special attention must be paid to explain these simulated data. Secondly, although the *I*_*Kur*_ model is based on that of Maleckar *et al*.^58^, the width of the *I*_*Kur*_ peak in the TPA model is different from that in the Maleckar *et al*. model^58^ and the Grandi *et al*. model^40^ because of the spike-and-dome-type action potential of our and CRN models (Supplementary Fig. S3). Thirdly, the APD restitution curve of the TPA model is similar to that of the TP ventricular model and it goes down more sharply than atrial restitution curves observed in experiments when approaching fast pacing rates, though it is within the experimental range of atrial cells (Fig. 7) and leads to a stable spiral wave (Supplementary Fig. S9 and Video S1). In comparison, the drop-off of other atrial restitution curves happens at even slower pacing rates and reduces more gradually. Fourthly, due to the lack of a precise model of complex calcium cycling in the TPA model, this study does not consider the effects of dantrolene on calcium sparks and further refinement of the model is required. Future improvement will need to include more accurate representations of the calcium handling of the human atrial cellular kinetics. In this study, the calcium handling of the TPA model displays an upstroke that coincides with the AP upstroke and shows a prolongated plateau phase, similar to that of ventricular models (Fig. 7(a,b)). The CRN_TP model improves the plateau phase in calcium concentration, however, calcium concentration still increases too fast (Supplementary Fig. S8a). While typical human atrial models, such as Grandi *et al*. and CRN models, have an upstroke in calcium concentration that happens after some delay from the upstroke of AF and show a narrow peak. Fifthly, the model does not represent atrial cells from the majority of patients with AF, instead it is designed specifically to study left atrial cells from patients with TBX5/PITX2 insufficiency. Finally, connexins (i.e., GJA1and GJA5) critical to atrial conduction are significantly down-regulated, and atrial conduction and reentrant arrhythmias at the organ level were observed in TBX5 insufficiency mouse atria^21^. Although this study focuses on the cellular basis of atrial arrhythmogenesis, the initiation and maintenance of AF at the tissue level should be further investigated in future. Nevertheless, whilst it is important to make explicit the potential limitations of the approaches adopted in the present study, these potential limitations are not expected to influence fundamental conclusions that can be drawn from our data on the mechanisms by which suppressed *I*_*K1*_ and inward *I*_*NCX*_ due to increased SR calcium leak contribute to the generation of spontaneous depolarizations in human atrial cells of homozygous TBX5-knockout atria.

In conclusion, we have demonstrated that electrical remodeling induced by TBX5 insufficiency causes disrupted SR calcium handling, AP prolongation and spontaneous depolarizations in human atrial cells. For the first time, we propose a novel cellular mechanism underlying TBX5 insufficiency induced-AF: that the imbalance between suppressed *I*_*K1*_ and inward *I*_*NCX*_ due to increased SR calcium leak causes spontaneous depolarizations in human atrial cells. More importantly, our study suggests that these arrhythmogenic triggers can be potently suppressed by inhibiting SR calcium leak or reversing remodeled *I*_*K1*_. Therefore, the outcomes of this study may lead to more targeted patient-specific treatment for AF in the near future.

## Methods

### Human atrial cell model

For this study, a new human atrial cellular model *alias* TPA was developed based on the TP model of ventricular epicardial cells. The TP model was chosen as the base model for modification as it incorporates human cell data and is also able to reproduce human AP morphology, APD rate dependence and detailed calcium handling^37^. The RyR channel model of the TP model was further modified with the combination of calcium-induced-calcium release flow *J*_*cicr*_ and SR calcium leak flow *J*_*leak*_^44,45^ to reproduce triggered activity, i.e., EAD, DAD and spontaneous depolarizations. In our study, the SR calcium flow (*J*_*rel*_) of RyR is given by

1 $${J}_{rel}={J}_{cicr}+{J}_{leak}$$

2 $${J}_{cicr}={V}_{rel}\cdot O\cdot ({[C{a}^{2+}]}_{SR}-{[C{a}^{2+}]}_{SS})$$

3 $${J}_{leak}={V}_{sp}\cdot R\cdot ({[C{a}^{2+}]}_{SR}-{[C{a}^{2+}]}_{SS})$$

where *V*_*rel*_ (0.102 mM/ms) is the maximal *J*_*cicr*_ conductance, *V*_*sp*_ (0.00036 mM/ms) is the maximal *J*_*leak*_ conductance, *O* is the proportion of open RyR channels, *R* is the proportion of closed RyR channels, [*Ca*^2+^]_SR_ is the free SR calcium concentration and [*Ca*^2+^]_SS_ is the free dyadic subspace calcium concentration^59^.

The TP model was then adjusted to generate our human atrial cell model TPA by incorporating experimentally documented ionic differences between human atrial and ventricular cells^40,41^. These modifications were based on most recent experimental data from human (wherever data was available) and from animals (where human data was unavailable), and our approach is consistent with those used in previous modeling studies^40,41^. Briefly, the maximal conductance of *I*_*CaL*_^40,41^, *I*_*to*_^41,60^, *I*_*K1*_^41,60^, *I*_*Ks*_, *I*_*Kr*_, *I*_*NCX*_^61^, sodium-potassium pump current (*I*_*NaK*_)^61^ and *J*_*up*_^40,62^ were modified (see Supplementary Table S1 for details). In addition to modifications of the parameters used in the TP model, the TPA model also incorporated the *I*_*Kur*_ current^58^.

4 $${I}_{Kur}={G}_{Kur}\cdot {X}_{Kur}\cdot {Y}_{Kur}\cdot ({V}_{m}-{E}_{K})$$

where *G*_*Kur*_ is the maximal conductance, *V*_*m*_ is voltage and *E*_*K*_ is the equilibrium (Nernst) potential for potassium ion. Activation gate *X*_*Kur*_ and inactivation gate *Y*_*Kur*_ are given by

5 $$\frac{d{X}_{Kur}}{dt}=\frac{({X}_{Kur,\infty }-{X}_{Kur})}{{\tau }_{X}}$$

6 $$\frac{d{Y}_{Kur}}{dt}=\frac{({Y}_{Kur,\infty }-{Y}_{Kur})}{{\tau }_{Y}}$$

Here, *t* is the time variable. *X*_*Kur*,∞_ and *Y*_*Kur*,∞_ denote for the steady-state for activation and inactivation, respectively, and τ_*X*_/τ_*Y*_ is the time constant for *X*_*Kur*,∞_/*Y*_*Kur*,∞_. The mathematical expressions of *X*_*Kur*,∞_ and *Y*_*Kur*,∞_ are given by

7 $${X}_{Kur,\infty }=\frac{1.0}{1.0+{e}^{-({V}_{m}+6.0)/8.6}}$$

8 $${Y}_{Kur,\infty }=\frac{1.0}{1.0+{e}^{({V}_{m}+7.5)/10.0}}$$

9 $${\tau }_{X}=\frac{9.0}{1.0+{e}^{({V}_{m}+5.0)/12.0}}+0.5$$

10 $${\tau }_{Y}=\frac{590.0}{1.0+{e}^{({V}_{m}+60.0)/10.0}}+3050.0$$

Intracellular structure (Supplementary Fig. S1a), AP morphology and key underlying ionic channels (Supplementary Fig. S1b) of the TP model were compared side by side with those of the TPA model. There are some marked differences between the TPA model and the TP model. The TPA model has a higher RMP, a more negative notch and a less pronounced dome than the TP model. These differences can be attributed to the reduction of *I*_*K1*_, the introduction of *I*_*Kur*_, and the modifications to *I*_*CaL*_, *I*_*to*_, *I*_*Kr*_, *I*_*Ks*_, *I*_*Kur*_, *I*_*NaK*_ and *I*_*NCX*_, respectively. This model represents the left atrial cells. All supporting data and computer simulation source code (http://models.cellml.org/workspace/520) from this study are available in the Supplementary Materials.

The higher RMP in the TPA model occurs during phase 4 of AP where the reduction of *I*_*K1*_ elevates a resting potential in the TPA compared to TP (RMP of −77.6 mV versus −86.2 mV). During phase 0 of AP, sodium channels recover more slowly from inactivation, resulting in a smaller *I*_*Na*_ and a reduced maximal depolarization velocity (122.11 mV/ms) that is comparable to that measured in experiments of ~140 mV/ms^40,63^. Changes in *I*_*to*_ and *I*_*Kur*_ contribute to a more negative notch in the TPA (versus TP) model during the phase 1 of AP. During phases 2 and 3 of AP, the combination of *I*_*CaL*_ and potassium currents (e.g., *I*_*Kr*_, *I*_*Ks*_ and *I*_*Kur*_) in the TPA model generates a smaller net current, leading to a less pronounced dome. In addition to the higher RMP, the more negative notch, and the less pronounced dome, the TPA model also exhibits a shorter APD of 233.6 ms compared with that of the TP model.

### Validation of the TPA model

The TPA model was fully assessed by comparing with existing human atrial models, particularly in terms of APs and calcium transient kinetics, since they are essential to this study (Fig. 7). The [*Ca*^2+^]_i_ trace of the TPA model was comparable to that of Grandi *et al*. model^40^ (Fig. 7(a)), whereas the AP shape of the TPA model was similar to that of Courtemanche *et al*. model^42^ (Fig. 7(b)). The advantages of the Grandi *et al*. model and the Courtemanche *et al*. model are preserved in the developed TPA model. Due to the important role of calcium dynamics and APD rate dependence in AF initialization and maintenance, the calcium transient and APD_90_ restitution of all existing human atrial models were benchmarked here. For calcium transients (Fig. 7(c)), the calcium transient amplitude (*Ca*_*tran*_) and *Ca*_*diast*_ in the TPA model were similar to those (marked with stars) reported in the Grandi *et al*. model and the Koivumaki *et al*. model^64^ which are both known for their more accurate calcium dynamics. In details, *Ca*_*diast*_ (0.138 µM) and *Ca*_*tran*_ (0.498 µM) obtained from the TPA model were similar to the measured data from Neef *et al*.^35^ (*Ca*_*diast*_ = ~0.1566 µM) and Grandi *et al*.^40^ (*Ca*_*tran*_ = ~0.468 µM). In terms of APD rate dependence (Fig. 7(d)), the simulated APD_90_ lied within the range of experimentally measured data (•) by Bosch *et al*.^43^ where the APD_90_ restitution curve of the TPA model was compared to that of the Koivumaki *et al*. model. All these comparison results have demonstrated that the TPA model can reliably reproduce the key properties of human atrial myocytes.

### Electrophysiological modeling of TBX5/PITX2 insufficiency

**Figure 8:**
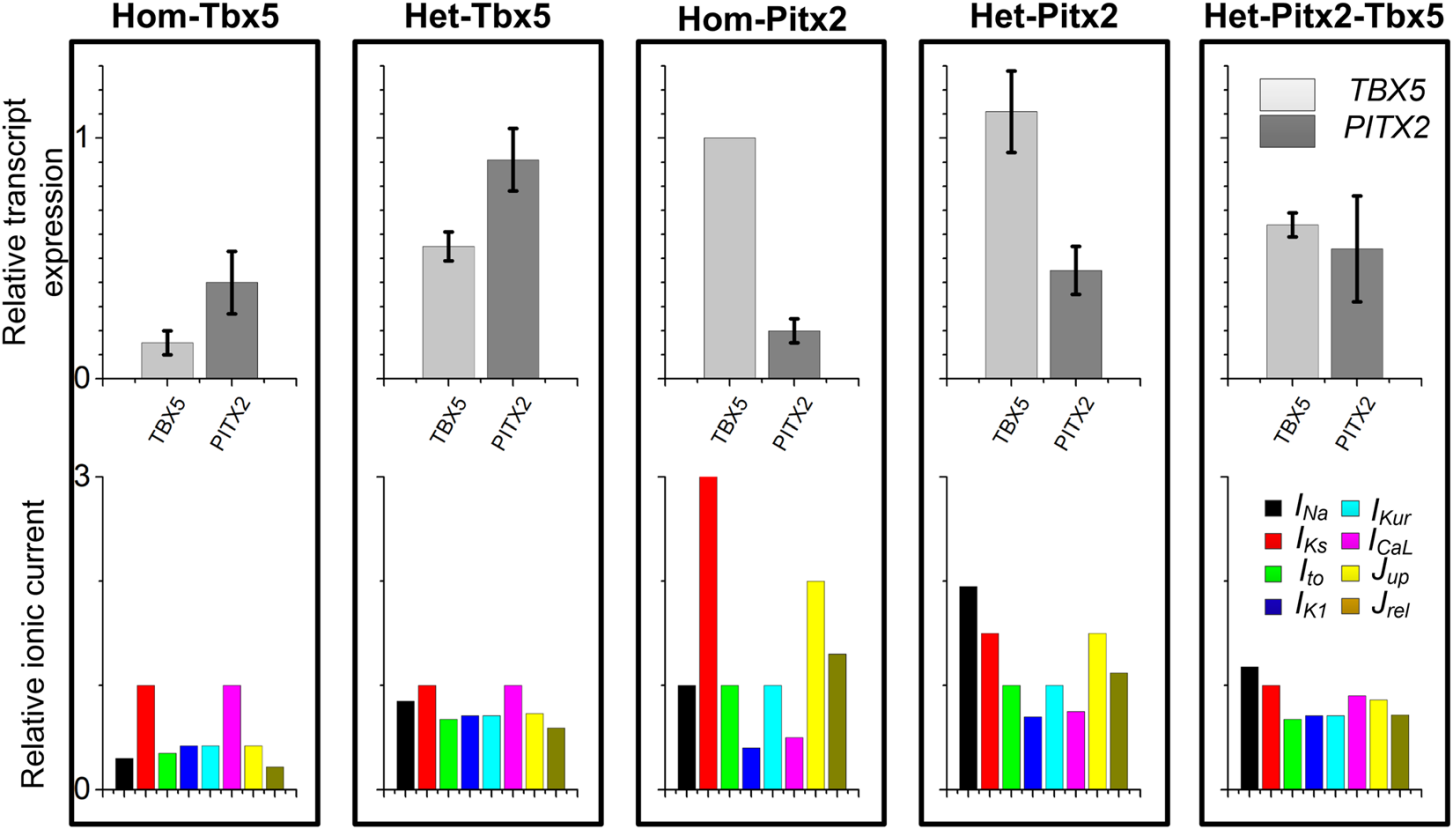
Ionic current changes in homozygous TBX5-knockout (Hom-Tbx5), heterozygous TBX5-knockout (Het-Tbx5), homozygous PITX2-knockout (Hom-Pitx2), heterozygous PITX2-knockout (Het-Pitx2), and heterozygous knockout of both PITX2 and TBX5 (Het-Pitx2-Tbx5) atrial cells. Relative transcript expression of TBX5 and PITX2, and relative ionic current of *I*_*Na*,_ *I*_*Ks*_, *I*_*to*_, *I*_*K1*_, *I*_*Kur*_, *I*_*CaL*_, *J*_*up*_ and *J*_*rel*_ in each condition.

According to changes in gene expression of TBX5/PITX2 in experiments^21,54,56,65^, five different scenarios were considered: Hom-Tbx5, Het-Tbx5, Hom-Pitx2, Het-Pitx2 and Het-Pitx2-Tbx5 conditions. For Hom-Pitx2 and Het-Pitx2 models, the relative transcript expression of TBX5 was not changed significantly (100% versus 111 ± 17%)^21,54^, and these models were mainly used to investigate the pro-arrhythmic effects of PITX2 insufficiency in a dose-dependent manner (~20% versus ~45%). For Hom-Tbx5 and Het-Tbx5 models, the relative gene expression in TBX5 was ~15% and ~55%^21^ respectively, so effects of TBX5 insufficiency at a different scale on human atrial electrophysiology were examined. In addition, Nadadur *et al*. have demonstrated that TBX5 directly activates PITX2 and reduced TBX5 leads to decreased PITX2 expression^21^. The relative gene expression in PITX2 for Hom-Tbx5 was set at ~53%, whereas that for Het-Tbx5 was ~91%^21^. The Het-Pitx2-Tbx5 model was designed to investigate the role of TBX5/PITX2 interplay in human atrial electrophysiology. In the Het-Pitx2-Tbx5 model, the relative gene expression of PITX2 was set at ~54%, whereas the relative gene expression of TBX5 was ~64%^21^. Based on experimental data on TBX5/PITX2 insufficiency-induced changes in gene expression of ion channels (see Supplementary Table S2 for details), modifications to ionic models for each case were implemented to represent electrical remodeling in atrial cells. Specifically, changes in *I*_*Na*_, *I*_*to*_, *I*_*Kur*_, *I*_*K1*_, *J*_*up*_ and *J*_*rel*_ were identified in TBX5-knockout atrial cells^21^, whereas remodeling of *I*_*Na*_, *I*_*CaL*_, *I*_*Ks*_, *I*_*K1*_, *J*_*up*_ and *J*_*rel*_ were identified in PITX2-knockout atrial cells^54,56,65^. For each model, relative transcript expression of TBX5 and PITX2 and modifications to ionic currents were described (see Fig. 8 for details).

## Electronic supplementary material

Supplementary Video_S1

Supplementary Video_S2

Supplementary Video_S3

Supplementary Information
