# Supplementary material for: Ionic and cellular mechanisms underlying TBX5/PITX2 insufficiency-induced atrial fibrillation: Insights from mathematical models of human atrial cells
Source: Sci Rep. 2018 Oct 23;8:15642. doi: 10.1038/s41598-018-33958-y (PMC6199257; doi:10.1038/s41598-018-33958-y)
Supplement: Supplementary file 4 — Supplementary Information [file 41598_2018_33958_MOESM4_ESM.pdf]

Ionic and cellular mechanisms underlying TBX5/PITX2 insufficiency-induced atrial fibrillation: Insights from mathematical models of human atrial cells

Jieyun Bai[1,5*], Patrick A. Gladding[2], Martin K. Stiles[3], Vadim V. Fedorov[4] & Jichao Zhao[1*]

[1] Auckland Bioengineering Institute, The University of Auckland, Auckland, New Zealand

[2] Department of Cardiology, Waitemata District Health Board, Auckland, New Zealand

[3] Waikato Hospital, Hamilton, New Zealand

[4] Department of Physiology and Cell Biology, The Ohio State University Wexner Medical Center, Columbus, United States of America

[5]School of Computer Science and Technology, Harbin Institute Technology, Harbin, China

[*]Correspondence and requests for materials should be addressed to J.Z. (j.zhao@auckland.ac.nz) or J.B. (jieyun.bai@auckland.ac.nz)

Methods

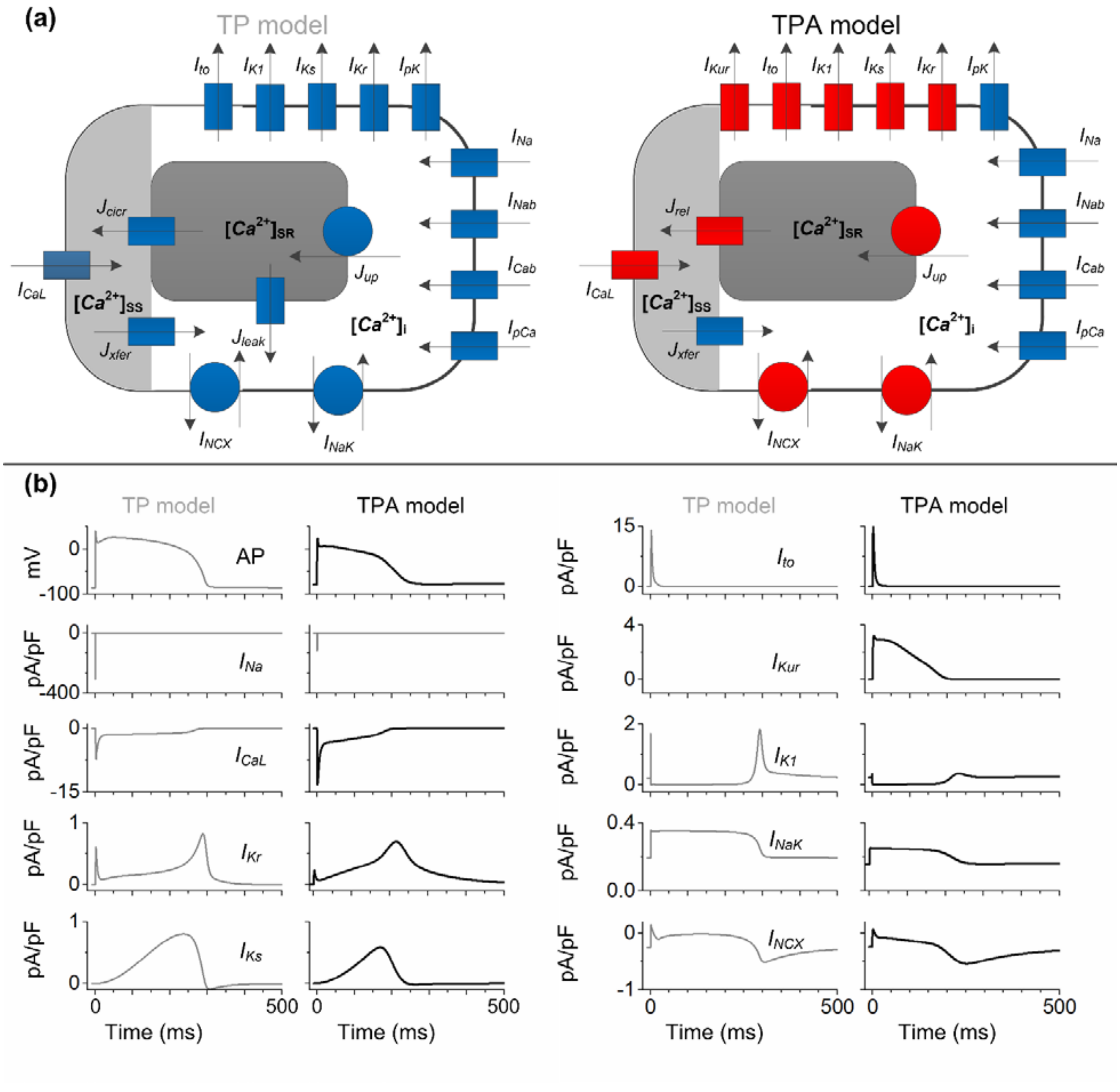

Supplementary Figure S1. Intracellular structure, action potential (AP) and major underlying currents for ventricular ten Tusscher-Panfilov (TP) model and our human atrial (TPA) model. (**a**) Outlines the main changes made to the TP model to generate the TPA model and schematic of the cell membrane, including the different modeled ionic currents and intracellular ion concentrations. L-type calcium current (I_{CaL}), rapid delayed rectifier potassium current (I_{Kr}), slow delayed rectifier potassium current (I_{Ks}), transient outward potassium current

(I_{to}), ultrarapid delayed rectifier potassium current (I_{Kur}), inward rectifier potassium current (I_{K1}), sodium-potassium pump current (I_{NaK}), sodium calcium exchange current (I_{NCX}) and the calcium flow (J_{up}) through the sarcoplasmic reticulum (SR) calcium ATPase (SERCA) and SR calcium release flow (J_{rel}) (including both calcium-induced-calcium release (J_{cicr}) and SR calcium leak (J_{leak})) are changed (marked in red). Fast sodium current (I_{Na}), background sodium current (I_{Nab}), background calcium current (I_{Cab}), plateau potassium current (I_{pK}), plateau calcium current (I_{pCa}) and diffusive calcium current (I_{xfer}) between dyadic subspace and bulk cytoplasm are not changed (marked in blue). Here, $[Ca^{2+}]_{SR}$ is the concentration of calcium ion in sub-cellular compartment SR (mM), $[Ca^{2+}]_{SS}$ is the concentration of calcium ion in sub-cellular compartment dyadic cleft (SS) and $[Ca^{2+}]_i$ is the cytosolic calcium concentration. (**b**) Steady-state action potential (AP) and the major underlying currents at a pacing frequency of 1Hz. Thinker traces represent voltage or currents for which maximal conductance or pump rate was changed to generate the TPA model from the TP model.

Supplementary Table S1. Differences between the utilized parameters in the ten Tusscher-Panfilov (TP) model and our human atrial (TPA) model.

Parameter	Definition	Value (TP)	Value (TPA)	Ref.
G_{CaL}	Maximal I_{CaL} conductance	0.0000398 cm·ms^{-1}·μF^{-1}	0.0000597 cm·ms^{-1}·μF^{-1}	Human [1,2]
G_{K1}	Maximal I_{K1} conductance	5.405 nS/pF	1.081 nS/pF	Human[2,3]
G_{to}	Maximal I_{to} conductance	0.294 nS/pF	0.588 nS/pF	Human[2,3]
G_{Kr}	Maximal I_{Kr} conductance	0.153 nS/pF	0.1989 nS/pF	⸸
G_{Ks}	Maximal I_{Ks} conductance	0.392 nS/pF	0.784 nS/pF	⸸
K_{NaK}	Maximal I_{NaK}	2.724 pA/pF	1.9068 pA/pF	Human [1,2,4]
K_{NCX}	Maximal I_{NCX}	1000 pA/pF	700 pA/pF	Human [1,2,4]
K_{sat}	Saturation factor for I_{NCX}	0.1	0.08	
K_{up}	Half-saturation constant of J_{up}	0.00025 mM	0.0005 mM	Human [1]

⸸ Values optimized to reproduce the best action potential (AP) shape.

Development of our human atrial model (TPA): The changes made to the ten Tusscher-Panfilov (TP)[5] model to generate our human atrial (TPA) model include alterations listed in the **Supplementary Table S1**, Maleckar et al. I_{Kur} [6] and modified calcium handling [7,8](see **Methods** for details). Intracellular structure (**Supplementary Fig. S1(a)**), AP morphology and key underlying ionic channels (**Supplementary Fig. S1(b)**) of the TP model were compared side by side to those of the TPA model.

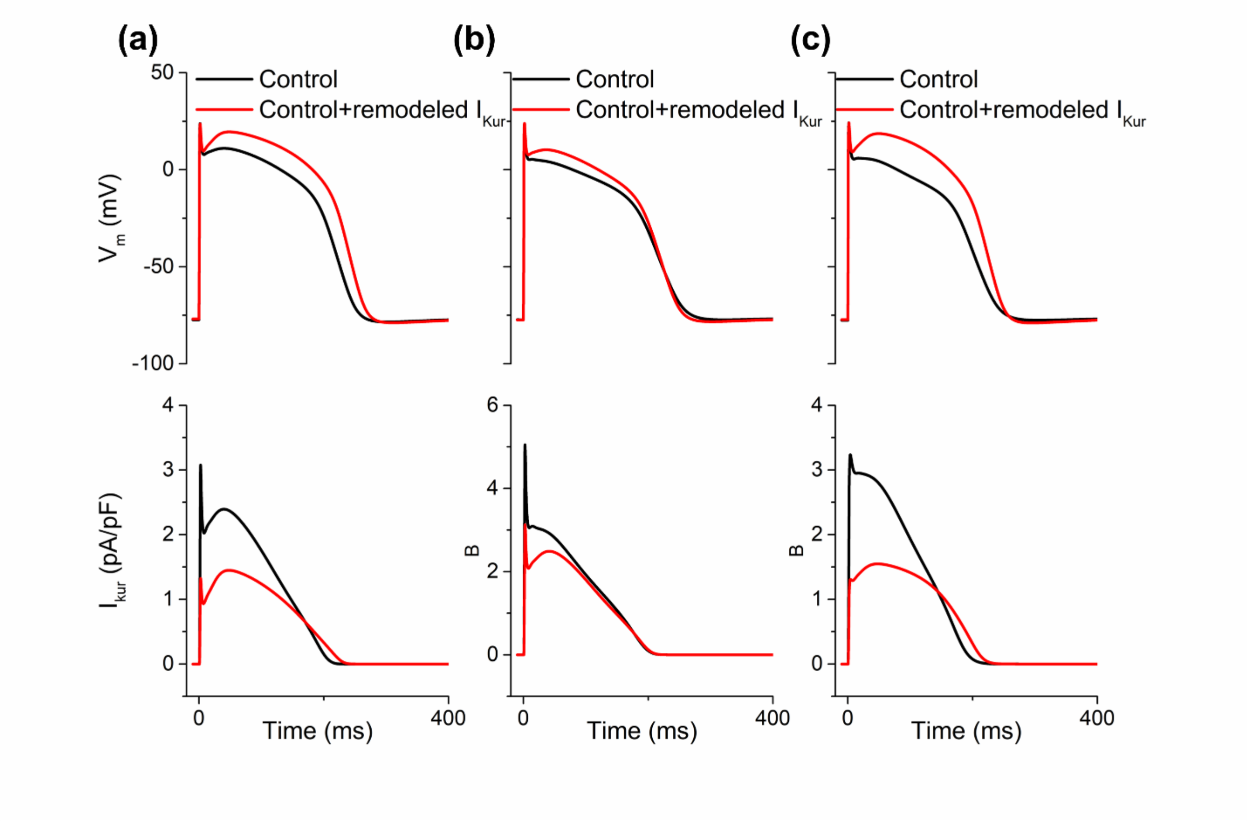

Supplementary Figure S2. Changes in action potential due to TBX5-induced I_{kur} remodeling using different formations of I_{kur}. Action potential and I_{kur} under control and remodeled I_{kur} conditions using I_{kur} of the Courtemanche et al. model (**a**), the Aguilar et al. model (**b)** and the Maleckar et al. model (c**)**. A reduction of I_{kur} due to TBX5 insufficiency led to prolongation of the atrial action potential.

Formulation of I_{Kur}**:** The I_{Kur} formulation in the TPA model was taken from the Maleckar et al.'s model[6]. In order to validate our TPA model, we also took into account all of the existing I_{Kur} models. These models include those adopted in previous simulation studies conducted by Courtemanche et al.[9], Aguilar et al. [10], and Maleckar et al.[6] Although different I_{kur} models were

used in our TPA model, it did not change the fact that reduction of I_{Kur} due to TBX5 insufficiency leads to prolongation of atrial action potentials (**Supplementary Fig. S2**). Equations and parameters for I_{Kur} in the Courtemanche et al. model are given by

$$\boldsymbol{X_{Kur,\infty} = \frac{1.0}{1.0 + e^{\frac{(V_m+30.3)}{-9.6}}}} \quad (1)$$

$$\boldsymbol{Y_{Kur,\infty} = \frac{1.0}{1.0 + e^{\frac{(V_m-99.45)}{27.48}}}} \quad (2)$$

$$\boldsymbol{\alpha_X = 0.65\left[e^{-\frac{(V_m+10)}{8.5}} + e^{-\frac{(V_m-30)}{59.0}}\right]^{-1}} \quad (3)$$

$$\boldsymbol{\beta_X = 0.65\left[2.5 + e^{\frac{(V_m+82)}{17.0}}\right]^{-1}} \quad (4)$$

$$\boldsymbol{\tau_X = [\alpha_X + \beta_X]^{-1}/K_{Q10}} \quad (5)$$

$$\boldsymbol{\alpha_Y = \left[1 + e^{-\frac{(V_m+30.3)}{9.6}}\right]^{-1}} \quad (6)$$

$$\boldsymbol{\beta_Y = \left[21.0 + e^{-\frac{(V_m-185.0)}{16.0}}\right]^{-1}} \quad (7)$$

$$\boldsymbol{\tau_Y = [\alpha_Y + \beta_Y]^{-1}/K_{Q10}} \quad (8)$$

$$\boldsymbol{G_{Kur} = 0.005 + \frac{0.05}{1.0 + e^{\frac{(V_m-15.0)}{-13}}}} \quad (9)$$

$$\boldsymbol{I_{Kur} = G_{Kur} \cdot X_{Kur} \cdot X_{Kur} \cdot X_{Kur} \cdot Y_{Kur} \cdot (V_m - E_K)} \quad (10)$$

Equations and parameters for I_{Kur} in the Aguilar et al. model are given by

$$\boldsymbol{X_{Kur,\infty} = \frac{1.0}{1.0 + e^{\frac{(V_m+30.3)}{-9.6}}}} \quad (11)$$

$$\boldsymbol{Y_{Kur,\infty} = \frac{1.0}{1.0 + e^{\frac{(V_m+5.0)}{5.0}}}} \quad (12)$$

$$Z_{Kur,\infty} = \frac{1.0}{1.0 + e^{\frac{(V_m - 35.0)}{20.0}}} \quad (13)$$

$$\alpha_X = 0.65\left[e^{-\frac{(V_m+10)}{8.5}} + e^{-\frac{(V_m-30)}{59.0}}\right]^{-1} \quad (14)$$

$$\beta_X = 0.65\left[2.5 + e^{\frac{(V_m+82)}{17.0}}\right]^{-1} \quad (15)$$

$$\tau_X = [\alpha_X + \beta_X]^{-1}/K_{Q10} \quad (16)$$

$$\tau_Y = 5800\left[1.0 + e^{-\frac{(V_m+80.0)}{11.0}}\right]^{-1} \quad (17)$$

$$\tau_Z = 800\left(2.0 - \frac{V_m}{40}\right) \quad (18)$$

$$G_{Kur} = 0.005 + \frac{0.05}{1.0 + e^{\frac{(V_m-15.0)}{-13}}} \quad (19)$$

$$I_{Kur} = G_{Kur} \cdot X_{Kur} \cdot X_{Kur} \cdot X_{Kur} \cdot Y_{Kur} \cdot Z_{Kur}(V_m - E_K) \quad (20)$$

Equations and parameters for I_{Kur} in the Maleckar et al. model are governed by

$$X_{Kur,\infty} = \frac{1.0}{1.0 + e^{-(V_m+6.0)/8.6}} \quad (21)$$

$$Y_{Kur,\infty} = \frac{1.0}{1.0 + e^{(V_m+7.5)/10.0}} \quad (22)$$

$$\tau_X = \frac{9.0}{1.0 + e^{(V_m+5.0)/12.0}} + 0.5 \quad (23)$$

$$\tau_Y = \frac{590.0}{1.0 + e^{(V_m+60.0)/10.0}} + 3050.0 \quad (24)$$

$$G_{Kur} = 0.045\ \mathrm{nS/pF} \quad (25)$$

$$I_{Kur} = G_{Kur} \cdot X_{Kur} \cdot Y_{Kur} \cdot (V_m - E_K) \quad (26)$$

Although the I_{Kur} model is based on that of Maleckar et al.'s model, the width of the I_{Kur} peak in the TPA model is different from that in the Maleckar et al. model. This discrepancy is likely due

to the different action potential types used between the TPA model (spike-and-dome-type action potential) and the Maleckar et al. model (spike-and-no-dome-type action potential). The action potential type between our TPA model and the CRN model is the same, as shown in the **Supplementary Fig. S3,** the action potential, I_{Kur} and I_{to} of the TPA model are similar to that of the CRN model. When spike-and-no-dome-type action potentials were created by blocking I_{CaL} in the TPA and CRN models, a resultant I_{Kur} similar to that of the Maleckar et al. model was obtained**.**

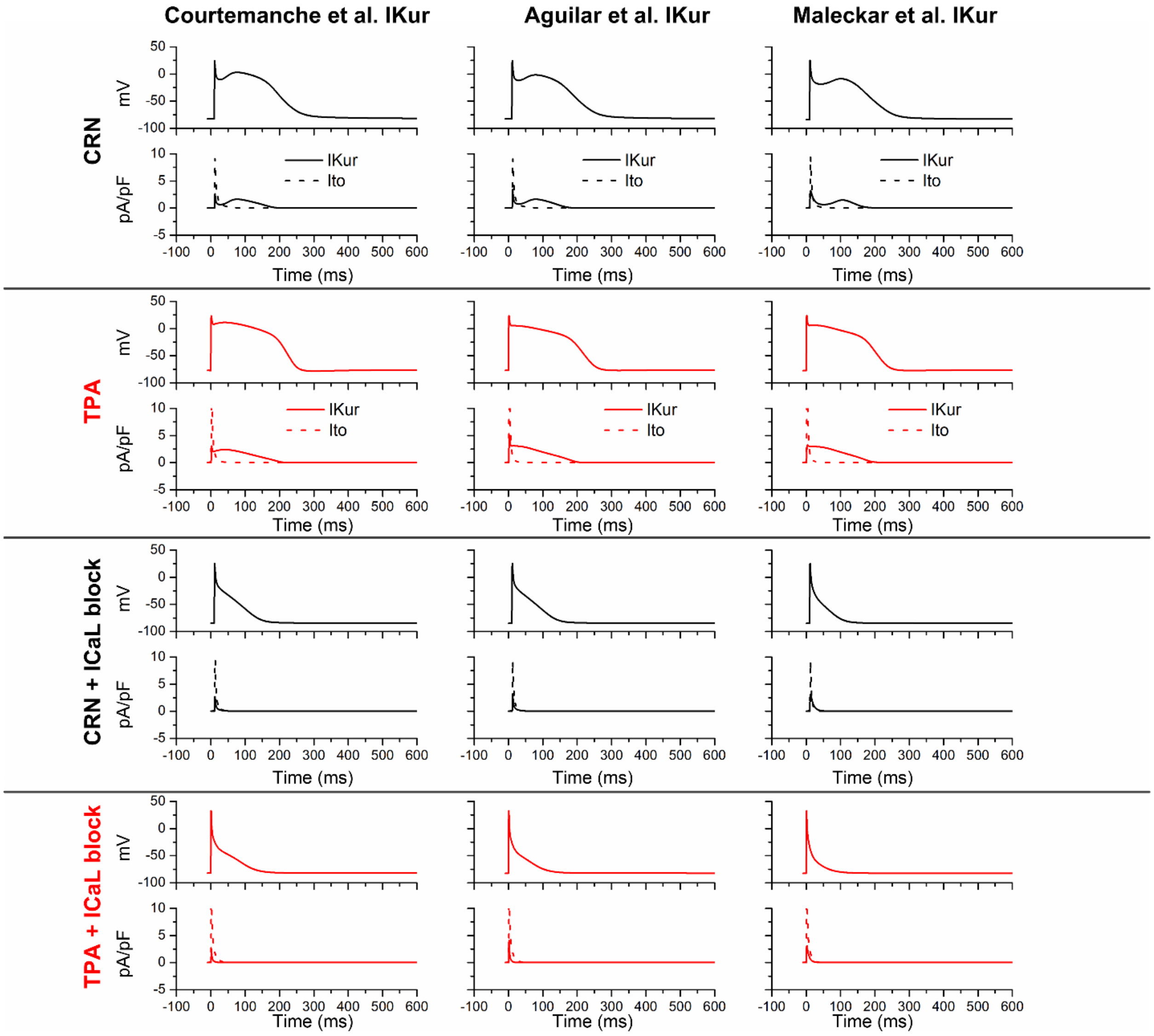

Supplementary Figure S3. Action potential, I_{Kur} and I_{to} of the CRN and TPA models, and the effects of I_{CaL}.

Electrophysiological modeling of TBX5/PITX2 insufficiency: According to changes in gene expression of TBX5/PITX2 observed in experiments, five different scenarios were considered: homozygous TBX5-knockout (Hom-Tbx5), heterozygous TBX5-knockout (Het-Tbx5), homozygous PITX2-knockout (Hom-Pitx2), heterozygous PITX2-knockout (Het-Pitx2) and heterozygous knockout of both PITX2 and TBX5 (Het-Pitx2-Tbx5) conditions. Alterations of ion currents relative to the TPA model can be found in the **Supplementary Table S2**

Supplementary Table S2. Review of atrial gene expression abnormalities induced by the Tbx5-Pitx2-dependent gene regulatory network

Process	Experimental observation				
	Hom-Tbx5	Hete-Tbx5	Hom-Pitx2	Hete-Pitx2	Hete-Tbx5-Pitx2
Tbx5 (%)	~15%[11]	~55%[11]	100%[12]	~111%[11]	~64%[11]
Pitx2 (%)	~53%[11]	~91%[11]	~200%[13], ~5%[14], ~10%-20%[15], 25%[16] and 20%[12]	~45%[11], ~30%[14] and ~35%[17]	~54%[11]
I_{Na} (%)	~30%[11]	~85%[11]	~40%[15]and ~50%[16]	~195%[11]	~118%[11]
I_{Ks} (%)	-	-	~200%[13] and ~300%[12]	-	-
I_{to} (%)	~35%[11]	-	-	-	-
I_{K1} (%)	~42%[11]	-	~40%[13]and ~130%[16]	-	-
I_{Kur} (%)	~42%[11]	-	-	-	-
I_{CaL} (%)	-	-	~53%[13]and ~50%[14]	~50%[14] and Decreased[17]	-
SERCA (%)	~42%[11]	~73%[11]	a major up regulation(~1100%)[14], 200%[16]and 150%[12]	~110%[11] and a major up regulation (~600%)[14]	~86%[11]
RyR (%)	~22%[11]	~59%[11]	a minor up regulation[14], 130%[16]and 300%[12]	~112%[11] and a minor up regulation[14]	~72%[11]

Validation of the TPA model for reproducing delayed afterdepolarization and spontaneous depolarization. To reproduce experimental observations, we simulated the three conditions: control (G_{K1}=5.405 nS/pF, V_{maxup}= 0.006375 mM/ms), control + increased SR Ca^{2+} (G_{K1}=5.405

nS/pF, V_{maxup}= 0.011475 mM/ms) and control + increased SR Ca^{2+}+ reduced I_{K1} (G_{K1}=1.081 nS/pF, V_{maxup}= 0.011475 mM/ms). Action potentials were elicited by the 50th stimulus at a cycle length of 250 ms. As shown in the **Supplementary Fig. S4,** increased SR Ca^{2+} (leading to DADs) and the reduced outward current I_{K1} contributed to diastolic depolarization, resulting in triggered action potentials.

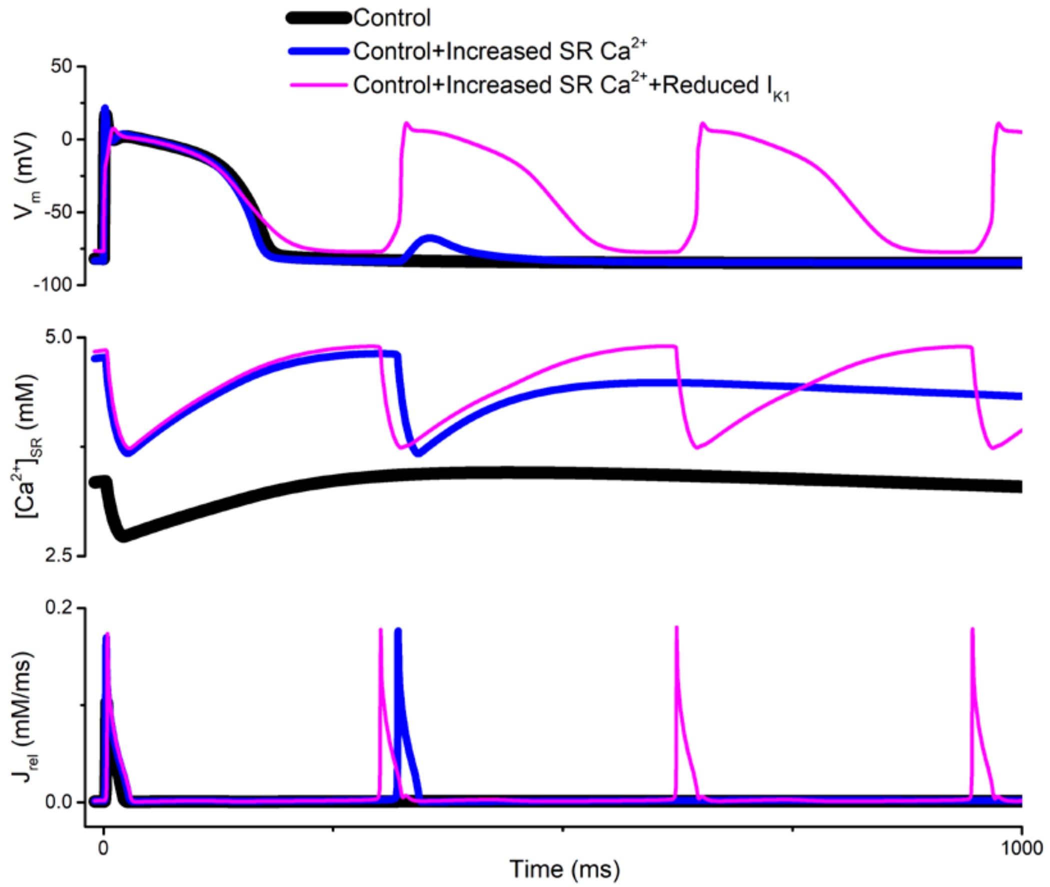

Supplementary Figure S4. Effects of increased sarcoplasmic reticulum (SR) Ca^{2+} and reduced I_{K1} on the membrane potential. Compared to the control condition (Black), increased SR Ca^{2+} leads to SR Ca^{2+} leak and delayed afterdepolarizations (DADs) (Blue). Reduced I_{K1} in addition to increased SR Ca^{2+} elevates resting membrane potential which favors the development of spontaneous depolarizations and DADs (Magenta).

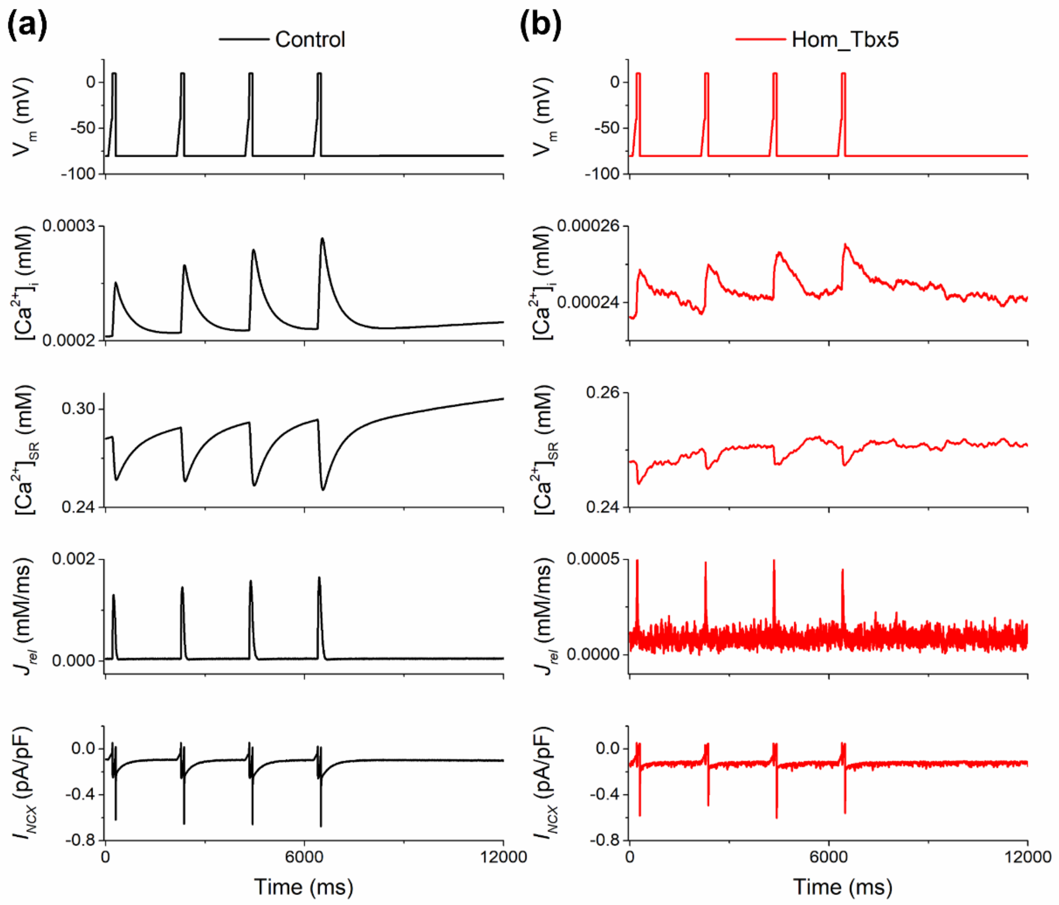

Supplementary Figure S5. **Electrophysiological properties under control and homozygous Tbx5-knockout (Hom_Tbx5) with 50 µmol/L flecainide conditions.** Compared to the control condition (**a**), the diastolic calcium concentration, spontaneous sarcoplasmic reticulum release events and activity of sodium-calcium exchangers were increased under the Hom_Tbx5 condition (**b**).

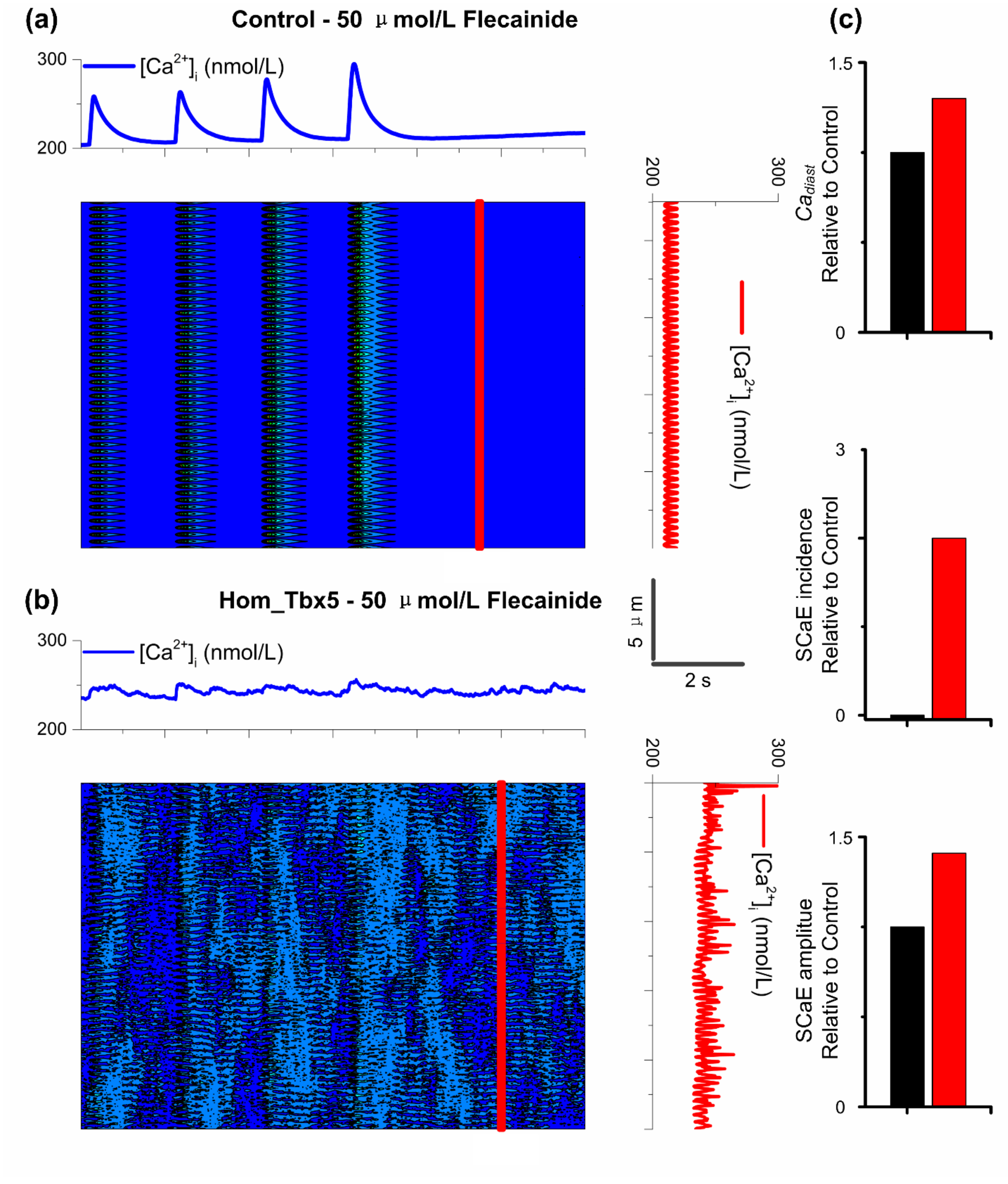

Supplementary Figure S6. **Spontaneous sarcoplasmic reticulum (SR)-release events (SCaEs) under control and homozygous tbx5-knockout (Hom_Tbx5) with 50 µmol/L flecainide conditions.** Transverse (Blue) and vertical (Red) line-scan representations of $[Ca^{2+}]_i$ for the control **a)** and Hom_Tbx5 **b)** models. **c)** The diastolic calcium concentration (Ca_{diast}) of SCaEs,

SCaEs incidence, and SCaEs amplitude were compared between control and Hom_Tbx5 conditions.

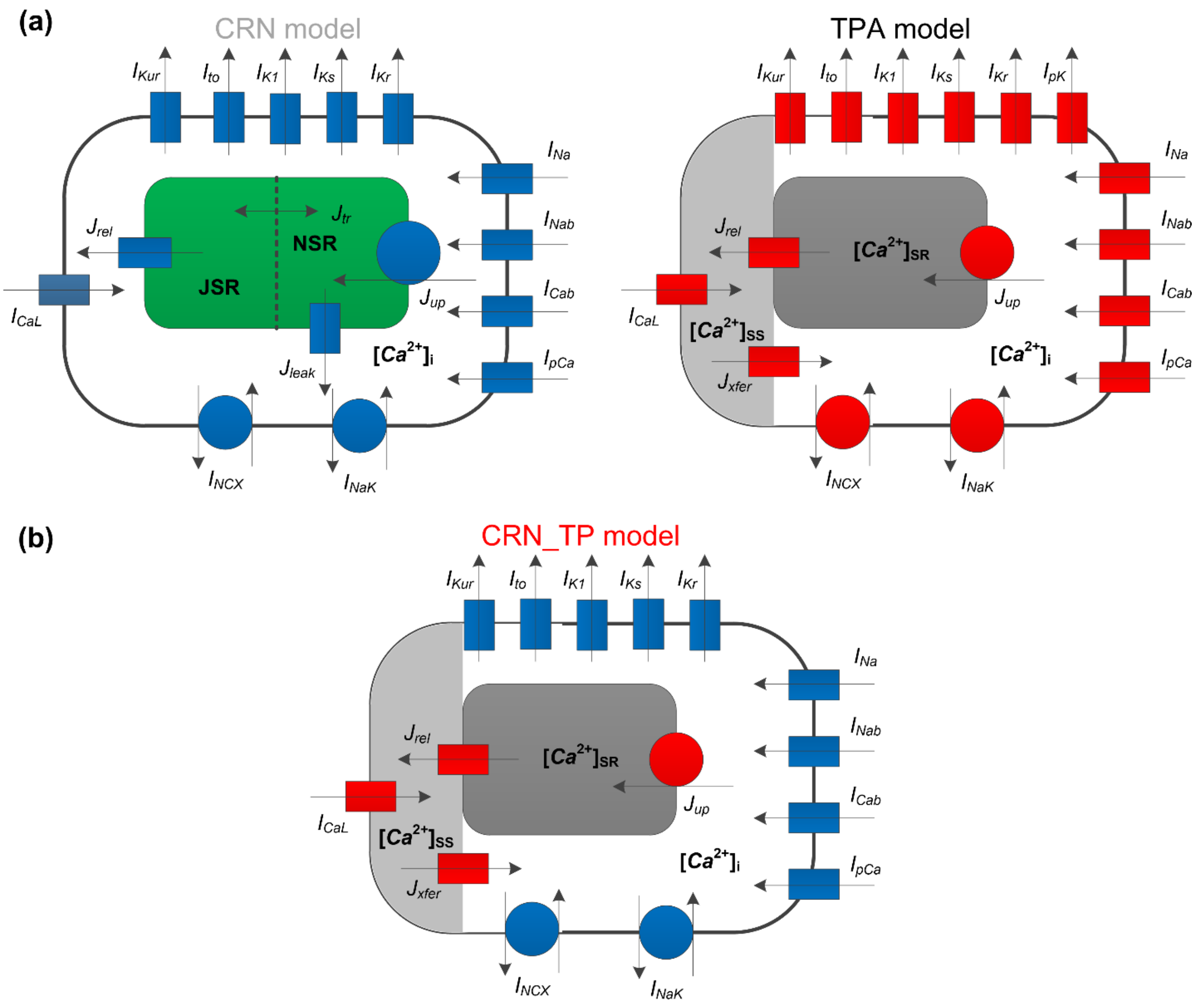

Supplementary Figure S7. Intracellular structures of the Courtemanche et al. model (CRN), our human atrial (TPA) model and a new human atrial model (CRN_TP) constructed by integrating the calcium dynamics of our TPA model into the CRN model. The intracellular structure and membrane ionic currents of the CRN (**a**) and TPA models (**b**). (**c**) The CRN_TP model was developed by combining the calcium handling formulations from the TPA model and the transmembrane currents of the CRN model. The cell space includes a sub-cellular compartment dyadic cleft (SS), the sarcoplasmic reticulum (SR), the cytoplasm and cell membrane.

Model independence of the cellular electrophysiological consequences of TBX5 insufficiency. In addition to our original TPA cell model, we also used the stochastic model[18] to test the single cell predictions for cases of TBX5 insufficiency. Under the control condition, spontaneous Ca^{2+}-release events (SCaEs) occurred and 50 μM/L flecainide could completely prevent these SCaEs[18]. To avoid the influence of these SCaEs, $[Ca^{2+}]_i$ was simulated under control and homozygous tbx5-knockout (Hom_Tbx5) with 50 μmol/L flecainide conditions. Our computer model was altered to simulate Hom_Tbx5 by adapting I_{Na} (-70%), I_{to} (-65%), I_{Kur} (-58%), I_{K1} (-58%), J_{up} (-58%), and J_{rel} (-78%) (see **Supplementary Table S2** for details). Action potentials were elicited at a cycle length of 2046 ms. The TBX5-induced electrical remodeling led to decreased systolic $[Ca^{2+}]_i$, increased diastolic calcium concentration (Ca_{diast}) and increased SR-Ca^{2+} leak (**Supplementary Fig. S5 and S6**), supporting our conclusion that increased SR-Ca^{2+} leak contributes to atrial arrhythmogenesis.

We also developed a new human atrial model (CRN_TP) by integrating the calcium dynamics of our TPA model into the Courtemanche et al. model (CRN). The intracellular structure of the CRN_TP model was compared to those of the CRN and TPA models (**Supplementary Fig. S7**). As shown in **Supplementary Fig. S8(a),** the action potential shape of the CRN_TP model was similar to that of the CRN model, but the amplitude of $[Ca^{2+}]_i$ in the CRN_TP model was larger than that of the CRN model.

To reproduce experimental observations, we simulated the three conditions: control (V_{maxup}= 0.006375 mM/ms), control + increased SR Ca^{2+} (V_{maxup}= 0.0082875 mM/ms) and control + increased SR Ca^{2+}+ reduced I_{K1} (80% reduction of G_{K1}, V_{maxup}= 0.0082875 mM/ms). Action potentials were elicited by the 50th stimulus at a cycle length of 500 ms. As shown in the **Supplementary Fig. S8,** the increased SR Ca^{2+} (leading to inward I_{NCX} and triggered action potential) and the reduced outward current I_{K1} contribute to diastolic depolarization, resulting in triggered action potentials.

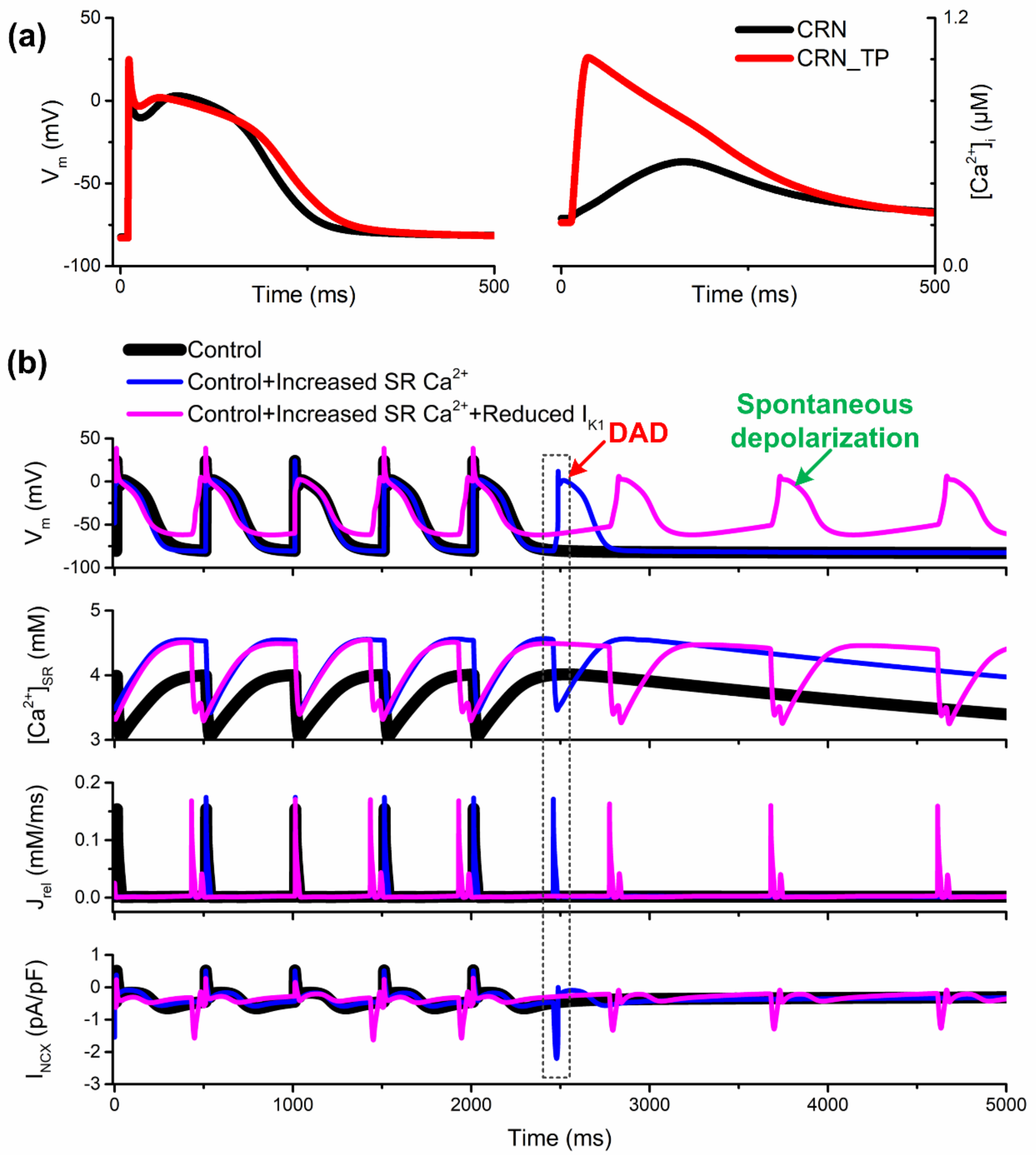

Supplementary Figure S8. Electrophysiological characteristics of the CRN_TP model. **(a)** Action potentials (left plane) and calcium transients (right plane) of the CRN (black) and CRN_TP (red) models. (**b**) Compared to the control condition, increased SR Ca^{2+} led to SR Ca^{2+} leak and delayed afterdepolarization (DAD). Further reduced I_{K1} elevated resting membrane potential, favoring the development of spontaneous diastolic depolarization.

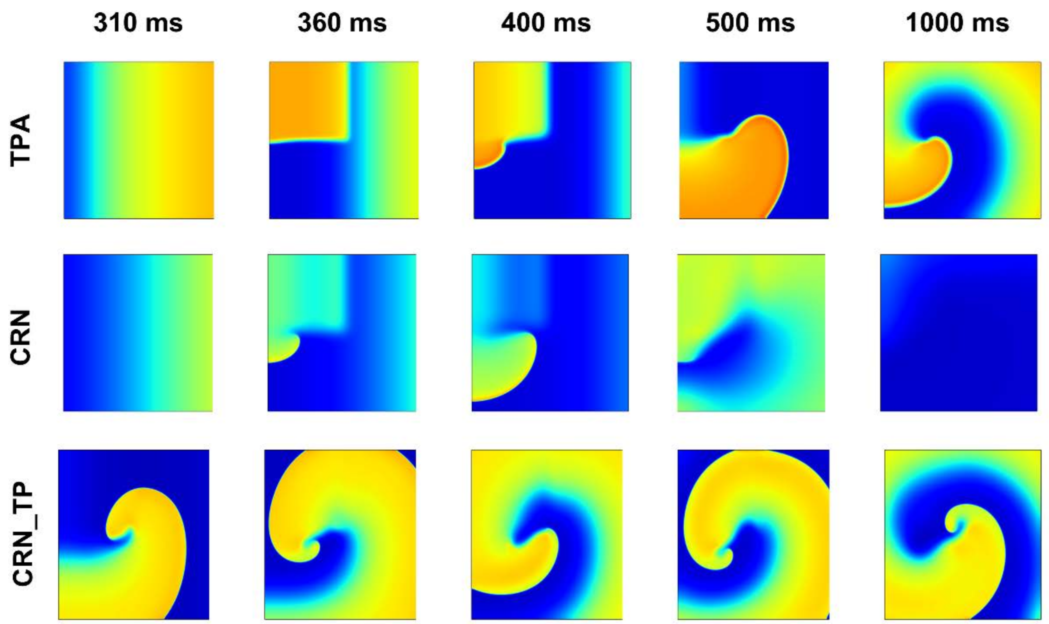

Supplementary Figure S9. Snapshots of initiation and conduction of re-entry in a 2D tissue using the TPA, CRN and CRN_TP models. Using our TPA and CRN_TP models, the spiral wave persisted. However, in the CRN model, the spiral wave self-terminated before t=1000ms.

Initiation of re-entry in a 2D sheet: Our two-dimensional (2D) simulation was carried out in an isotropic domain with D=0.1 mm^2/ms, a time step of 0.0025 ms and a space step of 0.15 mm in both the *x* and *y* directions. These 2D simulations have been performed on a domain of 500×500 grid points by using the explicit-Euler integration scheme. We have used Neumann no-flux boundary conditions. Re-entry was initiated by a conventional S1-S2 cross-field protocol. As shown in the **Supplementary Fig. S9,** stable spiral waves can be obtained using our TPA and CRN_TP models. In comparison, an unstable spiral wave was observed using the CRN model in our simulations which is similar to what was observed in other studies[19,20].

Supplementary Video S1: Reentry in an idealized 2D geometry using the TPA model. A re-entrant spiral wave was generated by the application of a premature S2 stimulus after a delay of 350 ms from the initial wave stimulus. The induced spiral wave persisted.

Supplementary Video S2: Reentry in an idealized 2D geometry using the CRN model. A re-entrant spiral wave was generated by the application of a premature S2 stimulus after a delay of 320 ms from the initial wave stimulus. The induced spiral wave self-terminated within 750 ms.

Supplementary Video S3: Reentry in an idealized 2D geometry using the CRN_TP model. A re-entrant spiral wave was generated by the application of a premature S2 stimulus after a delay of 190 ms from the initial wave. The induced spiral wave persisted.

References

1 Grandi, E. *et al.* Human Atrial Action Potential and Ca2+ Model Sinus Rhythm and Chronic Atrial Fibrillation. *Circ.Res.* **109**, 1055-1066, doi:10.1161/circresaha.111.253955 (2011).

2 Paci, M., Hyttinen, J., Aalto-Setälä, K. & Severi, S. Computational Models of Ventricular- and Atrial-Like Human Induced Pluripotent Stem Cell Derived Cardiomyocytes. *Ann Biomed Eng* **41**, 2334-2348, doi:10.1007/s10439-013-0833-3 (2013).

3 Amos, G. J. *et al.* Differences between outward currents of human atrial and subepicardial ventricular myocytes. *J Physiol* **491**, 31-50, doi:10.1113/jphysiol.1996.sp021194 (1996).

4 Wang, J. *et al.* Regional expression of sodium pump subunits isoforms and Na+-Ca++ exchanger in the human heart. *J Clin Invest* **98**, 1650-1658, doi:10.1172/JCI118960 (1996).

5 Tusscher, K. H. W. J. t. & Panfilov, A. V. Alternans and spiral breakup in a human ventricular tissue model. *Am J Physiol Heart Circ Physiol* **291**, H1088-H1100, doi:10.1152/ajpheart.00109.2006 (2006).

6 Maleckar, M. M., Greenstein, J. L., Giles, W. R. & Trayanova, N. A. K+ current changes account for the rate dependence of the action potential in the human atrial myocyte. *Am J Physiol Heart Circ Physiol* **297**, H1398-H1410, doi:10.1152/ajpheart.00411.2009 (2009).

7 Bai, J., Ren, Y., Wang, K. & Zhang, H. Mechanisms underlying the emergence of post-acidosis arrhythmia at the tissue level: A theoretical study. *Front Physiol* **8**, 195, doi:10.3389/fphys.2017.00195 (2017).

8 Bai, J. *et al.* Computational Cardiac Modeling Reveals Mechanisms of Ventricular Arrhythmogenesis in Long QT Syndrome Type 8: CACNA1C R858H Mutation Linked to Ventricular Fibrillation. *Front Physiol* **8**, 771, doi:10.3389/fphys.2017.00771 (2017).

9 Courtemanche, M., Ramirez, R. J. & Nattel, S. Ionic mechanisms underlying human atrial action potential properties: insights from a mathematical model. *Am J Physiol Heart Circ Physiol* **275**, H301-H321, doi:10.1152/ajpheart.1998.275.1.H301 (1998).

10 Aguilar, M., Feng, J., Vigmond, E., Comtois, P. & Nattel, S. Rate-dependent role of IKur in human atrial repolarization and atrial fibrillation maintenance. *Biophys J* **112**, 1997-2010, doi:10.1016/j.bpj.2017.03.022 (2017).

11 Nadadur, R. D. *et al.* Pitx2 modulates a Tbx5-dependent gene regulatory network to maintain atrial rhythm. *Sci Transl Med* **8**, 354ra115, doi:10.1126/scitranslmed.aaf4891 (2016).

12 Tao, Y. *et al.* Pitx2, an Atrial Fibrillation Predisposition Gene, Directly Regulates Ion Transport and Intercalated Disc Genes. *Circ Cardiovasc Genet* **7**, 23-32, doi:10.1161/circgenetics.113.000259 (2014).

13 Pérez-Hernández, M. *et al.* Pitx2c increases in atrial myocytes from chronic atrial fibrillation patients enhancing IKs and decreasing ICa,L. *Cardiovasc Res* **109**, 431-441, doi:10.1093/cvr/cvv280 (2016).

14 Lozano-Velasco, E. *et al.* Pitx2 impairs calcium handling in a dose-dependent manner by modulating Wnt signalling. *Cardiovasc Res* **109**, 55-66, doi:10.1093/cvr/cvv207 (2016).

15 Chinchilla, A. *et al.* PITX2 insufficiency leads to atrial electrical and structural remodeling linked to arrhythmogenesis. *Circ Genom Precis Med* **4**, 269-279, doi:10.1161/CIRCGENETICS.110.958116 (2011).

16 Lozano-Velasco, E. *et al.* Hyperthyroidism, but not hypertension, impairs PITX2 expression leading to Wnt-microRNA-ion channel remodeling. *PLoS One* **12**, e0188473, doi:10.1371/journal.pone.0188473 (2017).

17 Kirchhof, P. *et al.* PITX2c is expressed in the adult left atrium, and reducing Pitx2c expression promotes atrial fibrillation inducibility and complex changes in gene expression. *Circ Genom Precis Med* **4**, 123-133, doi:10.1161/Circgenetics.110.958058 (2011).

18 Voigt, N. *et al.* Cellular and molecular mechanisms of atrial arrhythmogenesis in patients with paroxysmal atrial fibrillation. *Circulation* **129**, 145-146, doi:10.1161/CIRCULATIONAHA.113.006641 (2013).

19 Cherry, E. M. & Evans, S. J. Properties of two human atrial cell models in tissue: restitution, memory, propagation, and reentry. *J Theor Biol* **254**, 674-690, doi:10.1016/j.jtbi.2008.06.030 (2008).

20 Wilhelms, M. *et al.* Benchmarking electrophysiological models of human atrial myocytes. *Front Physiol* **3**, 16, doi:10.3389/fphys.2012.00487 (2013).

```
//TPA.cpp
// Based on code by Jieyun Bai, Patrick A. Gladding, Martin K. Stiles, Vadim V. Fedorov,
Jichao Zhao
// Jieyun Bai v1.1  6 June 2018  jieyun.bai@auckland.ac.nz

// For more information
// see the inventors' web page at
// https://unidirectory.auckland.ac.nz/people/j-zhao

// Reference
// Ionic and cellular mechanisms underlying TBX5/PITX2 insufficiency-induced atrial
fibrillation: Insights from mathematical models of human atrial cells
// Jieyun Bai, Patrick A. Gladding, Martin K. Stiles, Vadim V. Fedorov &Jichao Zhao
// Scientific Reports. 2018.

//  This code is based on an earlier implementation of a similar model described in:
//  Bai, J. et al. Computational Cardiac Modeling Reveals Mechanisms of Ventricular
Arrhythmogenesis in Long QT Syndrome Type 8: CACNA1C R858H Mutation Linked to Ventricular
Fibrillation. Front Physiol. 8, 771.
//  Bai, J., Ren, Y., Wang, K. & Zhang, H. Mechanisms underlying the emergence of
post-acidosis arrhythmia at the tissue level: A theoretical study. Front Physiol. 8, 195.
//  Tusscher, K. H. W. J. t. and A. V. Panfilov (2006). "Alternans and spiral breakup in a
human ventricular tissue model." Am J Physiol Heart Circ Physiol. 291(3): H1088-H1100.


// Copyright (C) 2018, jieyun Bai
// All rights reserved.


// Redistribution and use in source and binary forms, with or without
// modification, are permitted provided that the following conditions
// are met:
//
//   1. Redistributions of source code must retain the above copyright
//      notice, this list of conditions and the following disclaimer.
//
//   2. Redistributions in binary form must reproduce the above copyright
//      notice, this list of conditions and the following disclaimer in the
//      documentation and/or other materials provided with the distribution.
//
//   3. The names of its contributors may not be used to endorse or promote
//      products derived from this software without specific prior written
//      permission.
//
// THIS SOFTWARE IS PROVIDED BY THE COPYRIGHT HOLDERS AND CONTRIBUTORS "AS IS"
// AND ANY EXPRESS OR IMPLIED WARRANTIES, INCLUDING, BUT NOT LIMITED TO, THE
// IMPLIED WARRANTIES OF MERCHANTABILITY AND FITNESS FOR A PARTICULAR PURPOSE
// ARE DISCLAIMED.  IN NO EVENT SHALL THE COPYRIGHT OWNER OR CONTRIBUTORS BE
// LIABLE FOR ANY DIRECT, INDIRECT, INCIDENTAL, SPECIAL, EXEMPLARY, OR
// CONSEQUENTIAL DAMAGES (INCLUDING, BUT NOT LIMITED TO, PROCUREMENT OF
// SUBSTITUTE GOODS OR SERVICES; LOSS OF USE, DATA, OR PROFITS; OR BUSINESS
// INTERRUPTION) HOWEVER CAUSED AND ON ANY THEORY OF LIABILITY, WHETHER IN
// CONTRACT, STRICT LIABILITY, OR TORT (INCLUDING NEGLIGENCE OR OTHERWISE)
// ARISING IN ANY WAY OUT OF THE USE OF THIS SOFTWARE, EVEN IF ADVISED OF THE
// POSSIBILITY OF SUCH DAMAGE.

// The original code included the following notice:
//
//     When you use this, send an email to: jieyun.bai@auckland.ac.nz
//     with an appropriate reference to your work.
//
// It would be nice to CC: j.zhao@auckland.ac.nz
// when you write.


// 06/06/18 added IKur equations for Ikur as in Maleckar et al. 2009
// 06/06/18 Changed IK1=*0.2
// 06/06/18 Changed Ito=*2
// 06/06/18 Changed IKs=*2
// 06/06/18 Changed IKr=*1.3
// 06/06/18 Changed INaK=0.7*
```

```
// 06/06/18 Changed INCX
// 06/06/18 Changed Jup
// 06/06/18 Changed Jrel
// 06/06/18 Changed ICaL=1.5*


#include <stdio.h>
#include <stdlib.h>
#include <math.h>

#define Begin                0
#define S1                   1000
#define S2                   1000
#define stim_strength        -80
#define stim_period          0.5
#define s1_beats             30
#define s2_beats             10
#define TRUE                 1
#define FALSE                0

#define HT                   0.02
#define HX                   0.2

#define WIDTH                1
#define LENGTH       (N_EPI+N_MCELL+N_ENDO+1)
#define N_EPI                40
#define N_MCELL              35
#define N_ENDO               25

#define ENDO                 1
#define MCELL                2
#define EPI                  3

#define HT                   0.02

#define DDVMT                0.154
#define DDVML                0.154

#define F                    96486.3415
#define T                    310.0
#define R                    8314.472

#define Vc               0.016404
#define Vsr              0.001094
#define Vss              0.00005468

#define inverseVcF2      (1./(2*Vc*F))
#define inverseVcF       (1./(Vc*F))
#define inversevssF2     (1./(2*Vss*F))
#define RTONF            (R*T/F)
#define CAPACITANCE      0.185

#define    PARAMETERS    25
#define    M        0
#define    H        1
#define    J        2
#define    Xr1      3
#define    Xr2      4
#define    Xs       5
#define    S        6
#define    R2       7
#define    D        8
#define    F1       9
#define    F2       10
#define    FCass    11
#define    RR       12
#define    OO       13
#define    Volt     14
#define    Volt2    15
#define    cai      16
#define    caSR     17
```

```
#define    caSS     18
#define    nai      19
#define    ki       20
#define    ssItot   21
#define    sxkur    22
#define    sykur    23
#define    sskur    24

#define    sm       paraVM[x][y][M]
#define    sh       paraVM[x][y][H]
#define    sj       paraVM[x][y][J]
#define    sxr1     paraVM[x][y][Xr1]
#define    sxr2     paraVM[x][y][Xr2]
#define    sxs      paraVM[x][y][Xs]
#define    ss       paraVM[x][y][S]
#define    sr       paraVM[x][y][R2]
#define    sd       paraVM[x][y][D]
#define    sf       paraVM[x][y][F1]
#define    sf2      paraVM[x][y][F2]
#define    sfcass   paraVM[x][y][FCass]
#define    sRR      paraVM[x][y][RR]
#define    sOO      paraVM[x][y][OO]
#define    svolt    paraVM[x][y][Volt]
#define    svolt2   paraVM[x][y][Volt2]
#define    Cai      paraVM[x][y][cai]
#define    CaSR     paraVM[x][y][caSR]
#define    CaSS     paraVM[x][y][caSS]
#define    Nai      paraVM[x][y][nai]
#define    Ki       paraVM[x][y][ki]
#define    sItot    paraVM[x][y][ssItot]
#define    xkur     paraVM[x][y][sxkur]  //Atrial
#define    ykur     paraVM[x][y][sykur]  //Atrial
#define    skur     paraVM[x][y][sskur]  //Atrial


const double Ko=5.4;
const double Cao=2.0;
const double Nao=140.0;

double  paraVM[WIDTH][LENGTH][PARAMETERS];
double  time=0;
int     filecounter=0;
int     origin[WIDTH][LENGTH];



double IKr=0;
double IKs=0;
double IK1=0;
double Ito=0;
double INa=0;
double IbNa=0;
double ICaL=0;
double ICaL_V=0;//11111111111111 jieyun
double IbCa=0;
double INaCa=0;
double IpCa=0;
double IpK=0;
double INaK=0;
double Irel=0;
double Ileak=0;//11111111111111 jieyun
double Irelease=0;
double Iup=0;
double Ixfer=0;
double I_kur=0;//  Atrial


int init()
{
    int x,y;
    for (x = 0; x < WIDTH; x++)
```

```
    {
        for (y = 0; y<LENGTH; y++)
        {
            paraVM[x][y][Volt]=-86.2;
            paraVM[x][y][Volt2]=-86.2;
            paraVM[x][y][cai] = 0.00007;
            paraVM[x][y][caSR] = 1.3;
            paraVM[x][y][caSS] = 0.00007;
            paraVM[x][y][nai] = 7.67;
            paraVM[x][y][ki] = 138.3;
            paraVM[x][y][M] = 0.0;
            paraVM[x][y][H] = 0.75;
            paraVM[x][y][J] = 0.75;
            paraVM[x][y][Xr1] = 0.0;
            paraVM[x][y][Xr2] = 1.0;
            paraVM[x][y][Xs] = 0.0;
            paraVM[x][y][R2] = 0.0;
            paraVM[x][y][S] = 1.0;
            paraVM[x][y][D] = 0.0;
            paraVM[x][y][F1] = 1.0;
            paraVM[x][y][F2] = 1.0;
            paraVM[x][y][FCass] = 1.0;
            paraVM[x][y][RR] = 1.0;
            paraVM[x][y][OO] = 0.0;
            paraVM[x][y][sxkur] = 3.824458e-04; //Atrial
            paraVM[x][y][sykur] = 9.597222e-01; //Atrial
            paraVM[x][y][sskur] = 9.597222e-01; //Atrial

        }
    }

    return 1;
}




double getVMItot(int x, int y, double dt, double Istim, int celltype)
{
    //Calcium buffering dynamics
    double Bufc=0.2;
    double Kbufc=0.001;
    double Bufsr=10.;
    double Kbufsr=0.3;
    double Bufss=0.4;
    double Kbufss=0.00025;

    //Intracellular calcium flux dynamics
    double Vmaxup=0.006375;
    double Kup=0.00025*2;//Atrial
    double Vrel=0.102;

    double k1_=0.15;
    double k2_=0.045;
    double k3=0.060;
    double k4=0.005;
    double EC=1.5;
    double maxsr=2.5;
    double minsr=1.;
    double Vleak=0.00036;
    double Vxfer=0.0038;

    double Gkr=0.153*1.3; //Atrial
    double pKNa=0.03;
    double GK1=5.405*0.2; //Atrial
    double GNa=14.838;
    double GbNa=0.00029;
    double KmK=1.0;
    double KmNa=40.0;
    double knak=2.724*0.7;//Atrial
```

```
    double GCaL=0.00003980*1.5;//Atrial
    double GbCa=0.000592;
    double knaca=1000*0.7;//Atrial
    double KmNai=87.5;
    double KmCa=1.38;
    double ksat=0.1*0.8;//Atrial
    double nn=0.35;
    double GpCa=0.1238;
    double KpCa=0.0005;
    double GpK=0.0146;

    double k1=0;
    double k2=0;
    double kCaSR=0;

    double dNai=0;
    double dKi=0;
    double dCai=0;
    double dCaSR=0;
    double dCaSS=0;
    double dRR=0;

    double Ek=0;
    double Ena=0;
    double Eks=0;
    double Eca=0;
    double CaCSQN=0;
    double bjsr=0;
    double cjsr=0;
    double CaSSBuf=0;
    double CaBuf=0;
    double bc=0;
    double cc=0;
    double Ak1=0;
    double Bk1=0;
    double rec_iK1=0;
    double rec_ipK=0;
    double rec_iNaK=0;
    double AM=0;
    double BM=0;
    double AH_1=0;
    double BH_1=0;
    double AH_2=0;
    double BH_2=0;
    double AJ_1=0;
    double BJ_1=0;
    double AJ_2=0;
    double BJ_2=0;
    double M_INF=0;
    double H_INF=0;
    double J_INF=0;
    double TAU_M=0;
    double TAU_H=0;
    double TAU_J=0;
    double axr1=0;
    double bxr1=0;
    double axr2=0;
    double bxr2=0;
    double Xr1_INF=0;
    double Xr2_INF=0;
    double TAU_Xr1=0;
    double TAU_Xr2=0;
    double Axs=0;
    double Bxs=0;
    double Xs_INF=0;
    double TAU_Xs=0;
    double R_INF=0;
    double TAU_R=0;
    double S_INF=0;
    double TAU_S=0;
    double Ad=0;
```

```
    double Bd=0;
    double Cd=0;

    double Af;
    double Bf;
    double Cf;
    double Af2;
    double Bf2;
    double Cf2;
    double bcss;
    double ccss;

    double TAU_D=0;
    double D_INF=0;
    double TAU_F=0;
    double F_INF=0;

    double TAU_F2;
    double F2_INF;
    double TAU_FCaSS;
    double FCaSS_INF;

    double xkur_INF;// Atrial
    double TAU_xkur;// Atrial
    double ykur_INF;// Atrial
    double TAU_ykur;// Atrial
    double GIkur;   // Atrial

    double Gks, Gto;
    Gks=0.392*2;  // Atrial
    Gto=0.294*2;  // Atrial

    Ek=RTONF*(log((Ko/Ki)));
    Ena=RTONF*(log((Nao/Nai)));
    Eks=RTONF*(log((Ko+pKNa*Nao)/(Ki+pKNa*Nai)));
    Eca=0.5*RTONF*(log((Cao/Cai)));

    Ak1=0.1/(1.+exp(0.06*(svolt-Ek-200)));
    Bk1=(3.*exp(0.0002*(svolt-Ek+100))+exp(0.1*(svolt-Ek-10)))/(1.+exp(-0.5*(svolt-Ek)));
    rec_iK1=Ak1/(Ak1+Bk1);

    rec_iNaK=(1./(1.+0.1245*exp(-0.1*svolt*F/(R*T))+0.0353*exp(-svolt*F/(R*T))));
    rec_ipK=1./(1.+exp((25-svolt)/5.98));

     // I_kur: Ultra rapid delayed rectifier Outward K Current
    GIkur =0.045;                               //Atrial
    I_kur = GIkur*xkur*ykur*(svolt-Ek);         //Atrial

    INa=GNa*sm*sm*sm*sh*sj*(svolt-Ena);

    ICaL=GCaL*sd*sf*sf2*sfcass*4*(svolt-15)*(F*F/(R*T))*(0.25*exp(2*(svolt-15)*F/(R*T))*CaSS-C
    ao)/(exp(2*(svolt-15)*F/(R*T))-1.);
    Ito=Gto*sr*ss*(svolt-Ek);
    IKr=Gkr*sqrt(Ko/5.4)*sxr1*sxr2*(svolt-Ek);
    IKs=Gks*sxs*sxs*(svolt-Eks);
    IK1=GK1*rec_iK1*(svolt-Ek);

    INaCa=knaca*(1./(KmNai*KmNai*KmNai+Nao*Nao*Nao))*(1./(KmCa+Cao))*(1./(1+ksat*exp((nn-1)*sv
    olt*F/(R*T))))*
        (exp(nn*svolt*F/(R*T))*Nai*Nai*Nai*Cao-exp((nn-1)*svolt*F/(R*T))*Nao*Nao*Nao*Cai*2.5);
    INaK=knak*(Ko/(Ko+KmK))*(Nai/(Nai+KmNa))*rec_iNaK;
    IpCa=GpCa*Cai/(KpCa+Cai);
    IpK=GpK*rec_ipK*(svolt-Ek);
    IbNa=GbNa*(svolt-Ena);
    IbCa=GbCa*(svolt-Eca);


    (sItot) = IKr    +IKs   +IK1   +Ito   +INa   +IbNa  +ICaL  +IbCa  +INaK  +INaCa +IpCa
    +IpK + I_kur +Istim;// Atrial

    kCaSR=maxsr-((maxsr-minsr)/(1+(EC/CaSR)*(EC/CaSR)));
```

```
    k1=k1_/kCaSR;
    k2=k2_*kCaSR;
    dRR=k4*(1-sRR)-k2*CaSS*sRR;
    sRR+=HT*dRR;
    sOO=k1*CaSS*CaSS*sRR/(k3+k1*CaSS*CaSS);

    Irel=Vrel*sOO*(CaSR-CaSS)+Vleak*sRR*(CaSR-CaSS);// Atrial
    Iup=Vmaxup/(1.+((Kup*Kup)/(Cai*Cai)));
    Ixfer=Vxfer*(CaSS-Cai);

    CaCSQN=Bufsr*CaSR/(CaSR+Kbufsr);
    dCaSR=HT*(Iup-Irel);                              //Atrial
    bjsr=Bufsr-CaCSQN-dCaSR-CaSR+Kbufsr;
    cjsr=Kbufsr*(CaCSQN+dCaSR+CaSR);
    CaSR=(sqrt(bjsr*bjsr+4*cjsr)-bjsr)/2;

    CaSSBuf=Bufss*CaSS/(CaSS+Kbufss);
    dCaSS=HT*(-Ixfer*(Vc/Vss)+Irel*(Vsr/Vss)+(-ICaL*inversevssF2*CAPACITANCE));
    bcss=Bufss-CaSSBuf-dCaSS-CaSS+Kbufss;
    ccss=Kbufss*(CaSSBuf+dCaSS+CaSS);
    CaSS=(sqrt(bcss*bcss+4*ccss)-bcss)/2;

    CaBuf=Bufc*Cai/(Cai+Kbufc);///////////
    dCai=HT*((-(IbCa+IpCa-2*INaCa)*inverseVcF2*CAPACITANCE)-(Iup)*(Vsr/Vc)+Ixfer); //Atrial
    bc=Bufc-CaBuf-dCai-Cai+Kbufc;
    cc=Kbufc*(CaBuf+dCai+Cai);
    Cai=(sqrt(bc*bc+4*cc)-bc)/2;

    dNai=-(INa+IbNa+3*INaK+3*INaCa)*inverseVcF*CAPACITANCE;
    Nai+=HT*dNai;

    dKi=-(Istim+IK1+Ito+IKr+I_kur+IKs-2*INaK+IpK)*inverseVcF*CAPACITANCE;         //Atrial
    Ki+=HT*dKi;

    AM=1./(1.+exp((-60.-svolt)/5.));
    BM=0.1/(1.+exp((svolt+35.)/5.))+0.10/(1.+exp((svolt-50.)/200.));
    TAU_M=AM*BM;
    M_INF=1./((1.+exp((-56.86-svolt)/9.03))*(1.+exp((-56.86-svolt)/9.03)));
    if (svolt>=-40.)
    {
        AH_1=0.;
        BH_1=(0.77/(0.13*(1.+exp(-(svolt+10.66)/11.1))));
        TAU_H= 1.0/(AH_1+BH_1);
    }
    else
    {
        AH_2=(0.057*exp(-(svolt+80.)/6.8));
        BH_2=(2.7*exp(0.079*svolt)+(3.1e5)*exp(0.3485*svolt));
        TAU_H=1.0/(AH_2+BH_2);
    }
    H_INF=1./((1.+exp((svolt+71.55)/7.43))*(1.+exp((svolt+71.55)/7.43)));

    if(svolt>=-40.)
    {
        AJ_1=0.;
        BJ_1=(0.6*exp((0.057)*svolt)/(1.+exp(-0.1*(svolt+32.))));
        TAU_J= 1.0/(AJ_1+BJ_1);
    }
    else
    {

        AJ_2=(((-2.5428e4)*exp(0.2444*svolt)-(6.948e-6)*exp(-0.04391*svolt))*(svolt+37.78)/(1.
        +exp(0.311*(svolt+79.23))));
        BJ_2=(0.02424*exp(-0.01052*svolt)/(1.+exp(-0.1378*(svolt+40.14))));
        TAU_J= 1.0/(AJ_2+BJ_2);
    }
    J_INF=H_INF;

    Xr1_INF=1./(1.+exp((-26.-svolt)/7.));
    axr1=450./(1.+exp((-45.-svolt)/10.));
    bxr1=6./(1.+exp((svolt-(-30.))/11.5));
```

```
    TAU_Xr1=axr1*bxr1;
    Xr2_INF=1./(1.+exp((svolt-(-88.))/24.));
    axr2=3./(1.+exp((-60.-svolt)/20.));
    bxr2=1.12/(1.+exp((svolt-60.)/20.));
    TAU_Xr2=axr2*bxr2;

    Xs_INF=1./(1.+exp((-5.-svolt)/14.));
    Axs=(1400./(sqrt(1.+exp((5.-svolt)/6))));
    Bxs=(1./(1.+exp((svolt-35.)/15.)));
    TAU_Xs=Axs*Bxs+80;


    R_INF=1./(1.+exp((20-svolt)/6.));
    S_INF=1./(1.+exp((svolt+20)/5.));
    TAU_R=9.5*exp(-(svolt+40.)*(svolt+40.)/1800.)+0.8;
    TAU_S=85.*exp(-(svolt+45.)*(svolt+45.)/320.)+5./(1.+exp((svolt-20.)/5.))+3.;

    D_INF=1./(1.+exp((-8-svolt)/7.5));
    Ad=1.4/(1.+exp((-35-svolt)/13))+0.25;
    Bd=1.4/(1.+exp((svolt+5)/5));
    Cd=1./(1.+exp((50-svolt)/20));
    TAU_D=(Ad*Bd+Cd);

    F_INF=1./(1.+exp((svolt+20)/7));

    Af=1102.5*exp(-(svolt+27)*(svolt+27)/225);
    Bf=200./(1+exp((13-svolt)/10.));
    Cf=(180./(1+exp((svolt+30)/10)))+20;
    TAU_F=(Af+Bf+Cf);
    F2_INF=0.67/(1.+exp((svolt+35)/7))+0.33;
    Af2=600*exp(-(svolt+25)*(svolt+25)/170);
    Bf2=31/(1.+exp((25-svolt)/10));
    Cf2=16/(1.+exp((svolt+30)/10));
    TAU_F2=(Af2+Bf2+Cf2);
    FCaSS_INF=0.6/(1+(CaSS/0.05)*(CaSS/0.05))+0.4;
    TAU_FCaSS=(80./(1+(CaSS/0.05)*(CaSS/0.05))+2.);

    xkur_INF=1./(1.+exp((svolt+6.)/-8.6));              //Atrial
    TAU_xkur=9./(1.+exp((svolt+5.)/12.0))+0.5;          //Atrial
    ykur_INF=1./(1.+exp((svolt+7.5)/10.));              //Atrial
    TAU_ykur=(590./(1.+exp((svolt+60.)/10.0))+3050.);   //Atrial


    sm = M_INF-(M_INF-sm)*exp(-HT/TAU_M);//111111111111111
    sh = H_INF-(H_INF-sh)*exp(-HT/TAU_H);//1111111111111
    sj = J_INF-(J_INF-sj)*exp(-HT/TAU_J);//111111111111111
    sxr1 = Xr1_INF-(Xr1_INF-sxr1)*exp(-HT/TAU_Xr1);//11111111111111
    sxr2 = Xr2_INF-(Xr2_INF-sxr2)*exp(-HT/TAU_Xr2);//111111111111
    sxs = Xs_INF-(Xs_INF-sxs)*exp(-HT/TAU_Xs);//111111111111
    ss= S_INF-(S_INF-ss)*exp(-HT/TAU_S);//11111111111111
    sr= R_INF-(R_INF-sr)*exp(-HT/TAU_R);//111111111
    sd = D_INF-(D_INF-sd)*exp(-HT/TAU_D);//111111111111
    sf =F_INF-(F_INF-sf)*exp(-HT/TAU_F);//1111111111111
    sf2 =F2_INF-(F2_INF-sf2)*exp(-HT/TAU_F2);//1111111111111
    sfcass =FCaSS_INF-(FCaSS_INF-sfcass)*exp(-HT/TAU_FCaSS);//11111111111111
    xkur = xkur_INF-(xkur_INF-xkur)*exp(-dt/TAU_xkur);//Atrial
    ykur = ykur_INF-(ykur_INF-ykur)*exp(-dt/TAU_ykur);//Atrial
    skur = 0;

            return (sItot);
}




void     Current_Contentions(){
    int celltype=EPI;
    int x=0,y=0;
    double time = 0;
    double tbegin = 0;
    double tend = 600;
```

```
    double stimtime=100;
    double Istim = 0;
    double dt = 0.02;
    double loadtime=600;
    int k = 0;
    int r = 0;
    int i = 0;
    int ii = 0;
    double t = 0;

    init();

    FILE * ap1,* ap2,* ap3,* ap4,* ap5,* ap6,* ap7,* ap8,* ap9,* ap10,* ap11,* ap12,* ap13,*
    ap14,* ap15,* ap16,* ap17,* ap18,* ap19,* ap20,* ap21,* ap22,* ap23,* ap24,* ap25,*
    ap26,* ap27,* ap28,* ap29,* ap30,* ap31,* ap32,* ap33,* ap34,* ap35;
    char * str1,* str2,* str3,* str4,* str5,* str6,* str7,* str8,* str9,* str10,* str11,*
    str12,* str13,* str14,* str15,* str16,* str17,* str18,* str19,* str20,* str21,* str22,*
    str23,* str24,* str25,* str26,* str27,* str28,* str29,* str30,* str31,* str32,* str33,*
    str34,* str35;

    str1 = (char*)malloc(32*sizeof(char));
    if (str1 == NULL){
        printf("\n out of memory ");
        exit(1);
    }
    sprintf(str1,"AP_%d.txt",celltype);
    printf("\n open the %s file", str1);
    ap1=fopen(str1,"w");
    if (ap1 == NULL){
        printf("\n cannot open file ");
        exit(1);
    }

   str2 =(char *) malloc(32*sizeof(char));
   if (str2 == NULL){
        printf("\n out of memory ");
        exit(1);
    }
    sprintf(str2," INa_%d.txt",celltype);
    ap2 = fopen(str2,"w");
    if (ap2 == NULL)
    {
        printf("\n cannot open file ");
        exit(1);
    }
    //free(str2);


 str4 =(char *) malloc(32*sizeof(char));
 if (str4 == NULL){
        printf("\n out of memory ");
        exit(1);
    }
   sprintf(str4," Ito_%d.txt",celltype);

    ap4 = fopen(str4,"w");
    if (ap4 == NULL)
    {
        printf("\n cannot open  file ");
        exit(1);
    }
    //free(str4);

str5 =(char *) malloc(32*sizeof(char));
if (str5 == NULL){
        printf("\n out of memory ");
        exit(1);
    }
    sprintf(str5," ICaL_%d.txt",celltype);

    ap5 = fopen(str5,"w");
```

```
    if (ap5 == NULL)
    {
        printf("\n cannot open  file ");
        exit(1);
    }
    //free(str5);

str6 =(char *) malloc(32*sizeof(char));
if (str6 == NULL){
        printf("\n out of memory ");
        exit(1);
    }
    sprintf(str6," OO_%d.txt",celltype);

    ap6 = fopen(str6,"w");
    if (ap6 == NULL)
    {
        printf("\n cannot open  file ");
        exit(1);
    }
    //free(str6);

str7 =(char *) malloc(32*sizeof(char));
if (str7 == NULL){
        printf("\n out of memory ");
        exit(1);
    }
    sprintf(str7," RR_%d.txt",celltype);

    ap7 = fopen(str7,"w");
    if (ap7 == NULL)
    {
        printf("\n cannot open  file ");
        exit(1);
    }
    //free(str7);

    str8 =(char *) malloc(32*sizeof(char));
if (str8 == NULL){
        printf("\n out of memory ");
        exit(1);
    }
    sprintf(str8," Jrel_%d.txt",celltype);

    ap8 = fopen(str8,"w");
    if (ap8 == NULL)
    {
        printf("\n cannot open  file ");
        exit(1);
    }
    //free(str8);

    str9 =(char *) malloc(32*sizeof(char));
if (str9 == NULL){
        printf("\n out of memory ");
        exit(1);
    }
    sprintf(str9," Jup_%d.txt",celltype);

    ap9 = fopen(str9,"w");
    if (ap9 == NULL)
    {
        printf("\n cannot open  file ");
        exit(1);
    }
    //free(str9);


    str10 =(char *) malloc(32*sizeof(char));
if (str10 == NULL){
        printf("\n out of memory ");
```

```
        exit(1);
    }
    sprintf(str10," IKr_%d.txt",celltype);

    ap10 = fopen(str10,"w");
    if (ap10 == NULL)
    {
        printf("\n cannot open  file ");
        exit(1);
    }
    //free(str10);


    str11 =(char *) malloc(32*sizeof(char));
if (str11 == NULL){
        printf("\n out of memory ");
        exit(1);
    }
    sprintf(str11," IKs_%d.txt",celltype);

    ap11= fopen(str11,"w");
    if (ap11 == NULL)
    {
        printf("\n cannot open  file ");
        exit(1);
    }
    //free(str11);


    str12 =(char *) malloc(32*sizeof(char));
if (str12 == NULL){
        printf("\n out of memory ");
        exit(1);
    }
    sprintf(str12," IK1_%d.txt",celltype);

    ap12= fopen(str12,"w");
    if (ap12 == NULL)
    {
        printf("\n cannot open  file ");
        exit(1);
    }
    //free(str12);


    str13 =(char *) malloc(32*sizeof(char));
if (str13 == NULL){
        printf("\n out of memory ");
        exit(1);
    }
    sprintf(str13," INaCa_%d.txt",celltype);

    ap13= fopen(str13,"w");
    if (ap13 == NULL)
    {
        printf("\n cannot open  file ");
        exit(1);
    }
    //free(str13);


    str14=(char *) malloc(32*sizeof(char));
if (str14 == NULL){
        printf("\n out of memory ");
        exit(1);
    }
    sprintf(str14," INaK_%d.txt",celltype);

    ap14 = fopen(str14,"w");
    if (ap14 == NULL)
    {
```

```
        printf("\n cannot open  file ");
        exit(1);
    }
    //free(str14);


    str15 =(char *) malloc(32*sizeof(char));
if (str15 == NULL){
        printf("\n out of memory ");
        exit(1);
    }
    sprintf(str15," IKb_%d.txt",celltype);

    ap15 = fopen(str15,"w");
    if (ap15 == NULL)
    {
        printf("\n cannot open  file ");
        exit(1);
    }
    //free(str15);


    str16 =(char *) malloc(32*sizeof(char));
if (str16 == NULL){
        printf("\n out of memory ");
        exit(1);
    }
    sprintf(str16," INab_%d.txt",celltype);

    ap16 = fopen(str16,"w");
    if (ap16 == NULL)
    {
        printf("\n cannot open  file ");
        exit(1);
    }
    //free(str16);


    str17 =(char *) malloc(32*sizeof(char));
if (str17 == NULL){
        printf("\n out of memory ");
        exit(1);
    }
    sprintf(str17," ICab_%d.txt",celltype);

    ap17 = fopen(str17,"w");
    if (ap17 == NULL)
    {
        printf("\n cannot open  file ");
        exit(1);
    }
    //free(str17);


    str18 =(char *) malloc(32*sizeof(char));
if (str18 == NULL){
        printf("\n out of memory ");
        exit(1);
    }
    sprintf(str18," IpCa_%d.txt",celltype);

    ap18 = fopen(str18,"w");
    if (ap18 == NULL)
    {
        printf("\n cannot open  file ");
        exit(1);
    }
    //free(str18);

        str19 =(char *) malloc(32*sizeof(char));
if (str19 == NULL){
```

```
        printf("\n out of memory ");
        exit(1);
    }
    sprintf(str19," Nai_%d.txt",celltype);

    ap19 = fopen(str19,"w");
    if (ap19 == NULL)
    {
        printf("\n cannot open  file ");
        exit(1);
    }
    //free(str19);


    str20 =(char *) malloc(32*sizeof(char));
if (str20 == NULL){
        printf("\n out of memory ");
        exit(1);
    }
    sprintf(str20," I_kur_%d.txt",celltype);

    ap20 = fopen(str20,"w");
    if (ap20 == NULL)
    {
        printf("\n cannot open  file ");
        exit(1);
    }
    //free(str20);


    str21 =(char *) malloc(32*sizeof(char));
if (str21 == NULL){
        printf("\n out of memory ");
        exit(1);
    }
    sprintf(str21," Ki_%d.txt",celltype);

    ap21= fopen(str21,"w");
    if (ap21 == NULL)
    {
        printf("\n cannot open  file ");
        exit(1);
    }
    //free(str21);

    str23 =(char *) malloc(32*sizeof(char));
if (str23 == NULL){
        printf("\n out of memory ");
        exit(1);
    }
    sprintf(str23," Cai_%d.txt",celltype);

    ap23= fopen(str23,"w");
    if (ap23 == NULL)
    {
        printf("\n cannot open  file ");
        exit(1);
    }
    //free(str23);


    str24=(char *) malloc(32*sizeof(char));
if (str24 == NULL){
        printf("\n out of memory ");
        exit(1);
    }
    sprintf(str24," Cass_%d.txt",celltype);

    ap24 = fopen(str24,"w");
    if (ap24 == NULL)
    {
```

```
        printf("\n cannot open  file ");
        exit(1);
    }
    //free(str24);


    str25 =(char *) malloc(32*sizeof(char));
if (str25 == NULL){
        printf("\n out of memory ");
        exit(1);
    }
    sprintf(str25," Casr_%d.txt",celltype);

    ap25 = fopen(str25,"w");
    if (ap25 == NULL)
    {
        printf("\n cannot open  file ");
        exit(1);
    }
    //free(str25);

    str32 =(char *) malloc(32*sizeof(char));
if (str32 == NULL){
        printf("\n out of memory ");
        exit(1);
    }
    sprintf(str32," Ixfer_%d.txt",celltype);

    ap32= fopen(str32,"w");
    if (ap32 == NULL)
    {
        printf("\n cannot open  file ");
        exit(1);
    }
    //free(str32);

    tbegin = Begin;
    time = 0;

    for (i = 1; i<=s1_beats; i++){
        if(i<50)
        tend = tbegin + S1;
        else
        tend = tbegin + S2;
        printf("\n S1.%d  - @: %f ms ", i, tend);
        while ( (tend-time) > dt*0.5 ){
            if((time>tbegin&&time<=(tbegin+stim_period)&&i<(s1_beats-5))) {
                Istim= stim_strength;
                printf ("start at: %f \n", time);
            }
            else
                Istim=0.0;

            stimtime = stimtime+dt;

            svolt = svolt - dt *  getVMItot(x, y, dt, Istim,celltype);

            time+=dt;

            if(time>=0&&time>(S1*(s1_beats-7)-10)&&time<((S1*(s1_beats-7)-10)+3000)){
            if (10 == k){
            k=0;
            fprintf(ap1,"%f %f ",time-S1*(s1_beats-7),svolt);
            fprintf(ap1," \n");
            fprintf(ap2,"%f %f ",time-S1*(s1_beats-7),INa);
            fprintf(ap2," \n");
            fprintf(ap4,"%f %f ",time-S1*(s1_beats-7),Ito);
            fprintf(ap4," \n");
            fprintf(ap5,"%f %f ",time-S1*(s1_beats-7),ICaL);
            fprintf(ap5," \n");
            fprintf(ap6,"%f %f ",time-S1*(s1_beats-7),sOO);
```

```
            fprintf(ap6," \n");
            fprintf(ap7,"%f %f ",time-S1*(s1_beats-7),sRR);
            fprintf(ap7," \n");
            fprintf(ap8,"%f %f ",time-S1*(s1_beats-7),Irel);
            fprintf(ap8," \n");
            fprintf(ap9,"%f %f ",time-S1*(s1_beats-7),Iup);
            fprintf(ap9," \n");
            fprintf(ap10,"%f %f ",time-S1*(s1_beats-7),IKr);
            fprintf(ap10," \n");
            fprintf(ap11,"%f %f ",time-S1*(s1_beats-7),IKs);
            fprintf(ap11," \n");
            fprintf(ap12,"%f %f ",time-S1*(s1_beats-7),IK1);
            fprintf(ap12," \n");
            fprintf(ap13,"%f %f ",time-S1*(s1_beats-7),INaCa);
            fprintf(ap13," \n");
            fprintf(ap14,"%f %f ",time-S1*(s1_beats-7),INaK);
            fprintf(ap14," \n");
            fprintf(ap15,"%f %f ",time-S1*(s1_beats-7),IpK);
            fprintf(ap15," \n");
            fprintf(ap16,"%f %f ",time-S1*(s1_beats-7),IbNa);
            fprintf(ap16," \n");
            fprintf(ap17,"%f %f ",time-S1*(s1_beats-7),IbCa);
            fprintf(ap17," \n");
            fprintf(ap18,"%f %f ",time-S1*(s1_beats-7),IpCa);
            fprintf(ap18," \n");
            fprintf(ap19,"%f %f ",time-S1*(s1_beats-7),Nai);
            fprintf(ap19," \n");
            fprintf(ap20,"%f %f ",time-S1*(s1_beats-7),I_kur);
            fprintf(ap20," \n");
            fprintf(ap21,"%f %f ",time-S1*(s1_beats-7),Ki);
            fprintf(ap21," \n");
            fprintf(ap23,"%f %f ",time-S1*(s1_beats-7),Cai);
            fprintf(ap23," \n");
            fprintf(ap24,"%f %f ",time-S1*(s1_beats-7),CaSS);
            fprintf(ap24," \n");
            fprintf(ap25,"%f %f ",time-S1*(s1_beats-7),CaSR);
            fprintf(ap25," \n");
            fprintf(ap32,"%f %f ",time-S1*(s1_beats-7),Ixfer);
            fprintf(ap32," \n");
            }
        k++;
            }
        }
        tbegin = tbegin + S1;
    }
     fclose(ap1);
     fclose(ap2);
     fclose(ap4);
     fclose(ap5);
     fclose(ap6);
     fclose(ap7);
     fclose(ap8);
     fclose(ap9);
     fclose(ap10);
     fclose(ap11);
     fclose(ap12);
     fclose(ap13);
     fclose(ap14);
     fclose(ap15);
     fclose(ap16);
     fclose(ap17);
     fclose(ap18);
     fclose(ap19);
     fclose(ap20);
     fclose(ap21);
     fclose(ap23);
     fclose(ap24);
     fclose(ap25);
     fclose(ap32);
}
```

```
int main()
{
    Current_Contentions();

    return 0;
}
```
